# Deciphering Photoinduced Charge Transfer Dynamics in a Cross-Linked Graphene–Dye Nanohybrid

**DOI:** 10.1021/acs.jpcc.1c10570

**Published:** 2022-02-16

**Authors:** Aaron M. Ross, Silvio Osella, Veronica R. Policht, Meng Zheng, Michele Maggini, Fabio Marangi, Giulio Cerullo, Teresa Gatti, Francesco Scotognella

**Affiliations:** † Department of Physics, Politecnico di Milano, Piazza Leonardo da Vinci 32, 20133 Milano, Italy; ‡ Chemical and Biological Systems Simulation Lab, Centre of New Technologies, University of Warsaw, Banacha 2C, 02-097 Warsaw, Poland; ¶ Chemical Sciences Department, Università degli Studi di Padova, Via Marzolo 1, 35131 Padova, Italy; § Center for Nano Science and Technology, Istituto Italiano di Tecnologia, Via Pascolo, 70/3 Milano 20133, Italy; ∥ Center for Materials Research, Justus Liebig University, Heinrich-Buff-Ring 17, 35392, Giessen, Germany

## Abstract

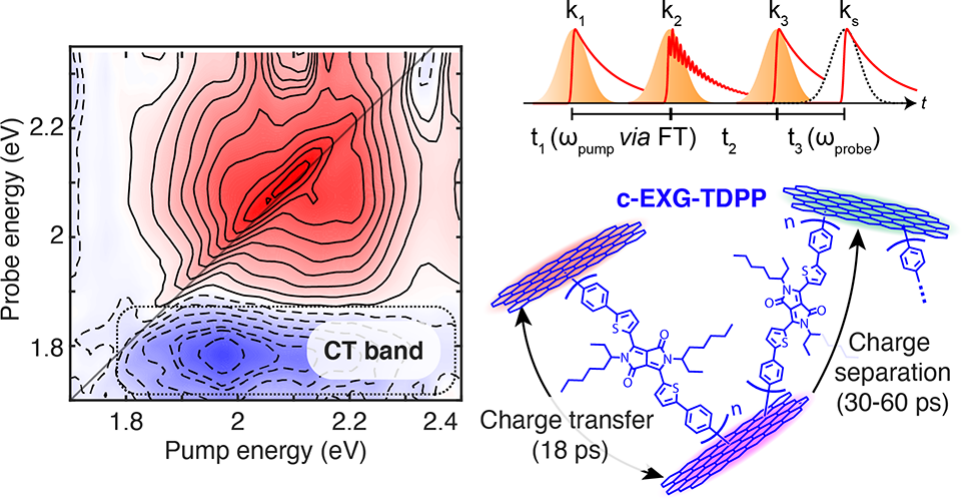

The search for synthetic materials that mimic natural photosynthesis by converting solar energy into other more useful forms of energy is an ever-growing research endeavor. Graphene-based materials, with their exceptional electronic and optical properties, are exemplary candidates for high-efficiency solar energy harvesting devices. High photoactivity can be conveniently achieved by functionalizing graphene with small molecule organic semiconductors whose band-gaps can be tuned by structural modification, leading to interactions between the π-conjugated electronic systems in both the semiconductor and graphene. Here we investigate the ultrafast transient optical properties of a cross-linked graphene–dye (diphenyl-dithiophenediketopyrrolopyrrole) nanohybrid material, in which oligomers of the organic semiconductor dye are covalently bound to a random network of few-layer graphene flakes, and compare the results to those obtained for the reference dye monomer. Using a combination of ultrafast transient absorption and two-dimensional electronic spectroscopy, we provide substantial evidence for photoinduced charge transfer that occurs within 18 ps in the nanohybrid system. Notably, subpicosecond photoinduced torsional relaxation observed in the constituent dye monomer is absent in the cross-linked nanohybrid system. Through density functional theory calculations, we compare the competing effects of covalent bonding, increasing conjugation length, and the presence of multiple graphene flakes. We find evidence that the observed ultrafast charge transfer process occurs through a superexchange mechanism in which the oligomeric dye bridge provides virtual states enabling charge transfer between graphene–dye covalent bond sites.

## Introduction

Photoinduced electron transfer is a key process in living photosynthetic systems that exploit energy from sunlight to promote transformation of simple molecules into useful chemical building blocks essential for their own survival.^1^ The electron transfer process in natural photosynthesis takes place with near unity quantum efficiency in reaction center pigment–protein complexes. The field of “artificial photosynthesis”^2^ focuses on achieving the same high performance of naturally occurring systems while improving upon their robustness and spectral coverage. Graphene-based materials (GBMs) are currently at the focus of one of the most concerted research efforts that the global scientific community has ever devoted to a single material platform.^3,4^ Many photoactive hybrids that integrate GBMs have been developed,^5,6^ with the aim to produce systems that convert light energy into electrical energy or solar fuels (solar cells, photoelectrochemical cells, photodetectors) but also for achieving intriguing luminescence properties or to carry out smart sensing. GBMs integrated with strong light-absorbing elements such as chromophores or nanoparticles have achieved many of these goals, where the arrangement of the graphene work function relative to the energy levels of the light-absorber lead to efficient transfer of excitation energy/charge to/from the GBM following photoexcitation. New examples of nanohybrids and nanocomposites based on GBMs and organic/organometallic chromophores are constantly appearing in the literature,^7−13^ indicative of the wide interest in the design, synthesis, photophysical characterization, and technological application of these species which will likely emerge in the future as key items in nanotechnology.

**Figure 1:**
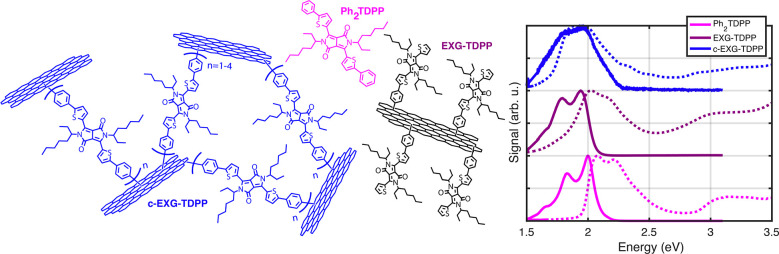
Molecular diagrams of systems under study, and linear absorption (dashed lines) and PL (solid lines) spectra of Ph_2_TDPP (pink), EXG–TDPP (purple), and c-EXG–TDPP (blue).

The two main strategies for assembling combinations of GBM units with light-harvesting units are via either covalent or noncovalent bonding. The latter strategy is generally based on the formation of π-stacking interactions between the chromophore and the GBM surface which allows for the conservation of the carbon nanostructure π-conjugated lattice. In contrast, the former introduces sp^3^ defects that locally interrupt the π-conjugation. One advantage of the covalent bonding strategy is the enhancement of stability of the nanohybrid, a necessary property for technological applications.^6,7,14,15^ We have recently reported^16^ on the synthesis of a novel covalent cross-linked nanohybrid, c-EXG–TDPP, which consists of oligomeric bifunctional diphenyl-dithiophenediketopyrrolopyrrole (Ph_2_TDPP) units that bridge few-layer exfoliated graphene flakes (EXG) to form a random polymeric structure (Figures 1 and S3). Compared to monomeric Ph_2_TDPP, the c-EXG–TDPP nanohybrid exhibits a very large red-shift in absorption of at least 60 nm and strong photoluminescence (PL) quenching. The presence of the oligomeric bridge structure in c-EXG–TDPP was confirmed by investigation of a non-cross-linking but otherwise analogous structure named EXG–TDPP.^16^

In this work, we investigate the strong electronic coupling between Ph_2_TDPP and EXG that results in the red-shift and PL quenching. We measure the electronic dynamics after photoexcitation in the c-EXG–TDPP nanohybrid and compare it to model systems Ph_2_TDPP and non-cross-linking EXG–TDPP via the combination of ultrafast transient absorption (TA), two-dimensional electronic spectroscopy (2DES), and density functional theory (DFT) calculations. We observe two sub-60 ps processes in the c-EXG–TDPP nanohybrid resolved via global analysis that correspond to ultrafast charge transfer (CT) and subsequent charge separation between the EXG and covalently bound TDPP units. These results explain the previously reported strong PL quenching in the nanohybrid.^16^ Additionally, we are able to attribute a subpicosecond dynamic Stokes shift in the Ph_2_TDPP monomer to torsional relaxation; this dynamic is noticeably absent in c-EXG–TDPP. DFT calculations provide evidence of charge transfer states in the c-EXG–TDPP system and suggest that this CT process likely proceeds via a superexchange mechanism between EXG–TDPP covalent bond sites mediated virtually by the oligomeric Ph_2_TDPP bridge. The combination of the observed ultrafast charge transfer due to a superexchange mechanism with the stability granted from the covalent linkage make the c-EXG–TDPP nanohybrid a promising system for future light-harvesting applications.

## Methods

### Materials Synthesis

All commercially available reagents and solvents were purchased from Sigma–Aldrich, Fluka, and TCI Chemicals and used as received. Graphene powder was purchased from Superior Graphite and used as received. The hybrids c-EXG–TDPP and EXG–TDPP as well as the small molecule Ph_2_TDPP were synthesized following the procedures described previously.^16^

### Linear Absorption and Steady-State PL

Linear absorption measurements were performed using a Jasco V-570 spectrophotometer. Steady-state PL experiments were performed using a nanosecond pulsed laser centered at 355 nm. The laser was focused with a 15 cm focal length lens onto a 1 mm path length cuvette containing each sample, and the PL was collected at 45° relative to the incident beam into a fiber and sent to a spectrometer. Sixty 1-s integrations were collected for each spectrum, with typical excitation average power of around 100 μW.

### Ultrafast Transient Absorption Spectroscopy

The laser system employed for the ultrafast transient absorption (TA) spectroscopy experiments is a Ti:sapphire chirped pulse amplifier (CPA) system that generates pulses with maximum energies of 800 μJ, pulse duration of around 100 fs, central wavelength of 800 nm, at a repetition rate of 1 kHz. The pump pulses are generated by seeding a noncollinear optical parametric amplifier (NOPA) with the attenuated output from the CPA; optical parametric amplification of narrowband filtered white light (10 nm bandwidth) in a β-barium borate (BBO) crystal pumped by the second harmonic 400 nm light yields pump pulses with tunable central wavelengths between 470 and 780 nm and pulse durations of 100 fs. For Ph_2_TDPP, EXG–TDPP, and c-EXG–TDPP experiments, the pulse central wavelengths were set to 575, 565, and 640 nm, respectively. Narrow bandwidth pulses were chosen to reduce the spectral range over which pump scattering obscured the signal. A broadband white light probe was generated by pumping a YAG crystal with focused 100 fs pulses centered at 1.24 μm produced in a separate nearly collinear OPA, with the 1.24 μm pump filtered after white light generation by a 1.05 μm short-pass filter. The broadband probe spectrum spanned from 530 to 1000 nm, with shot-to-shot noise less than 0.5%, and minimal leak-through from the fundamental CPA output at 800 nm. The pump pulses are mechanically modulated by a rotating chopper allowing every other pulse to pass (500 Hz pump pulse repetition rate) and are then polarized by transmission through a wire grid polarizer, with an arbitrary linear polarization chosen by transmission through a broadband half-wave plate; the polarization is chosen either cross or copolarized to the white light probe for anisotropy measurements. The pump–probe delay is varied by scanning a mechanical delay line with a retroreflector on the pump path out to at least 1 ns delay. The pump and probe pulses are focused and overlapped onto the sample at a small crossing angle, with spot diameters equal to 240 and 205 μm, respectively. Pump fluences at the sample are chosen between 9 and 310 μJ/cm^2^. Pump scattering is filtered by a wire grid polarizer set to transmit the probe, as well as spatial filtering via an iris after the sample. The transmitted probe is spectrally dispersed on a fast optical multichannel analyzer, which is synchronized to the repetition rate of the laser; differential transmission spectra Δ*T*/*T* =  are collected by comparing the sample-attenuated dispersed white light when the pump is incident on the sample or blocked.

### Two-Dimensional Electronic Spectroscopy

Two-dimensional electronic spectroscopy (2DES) was performed utilizing a partially noncollinear geometry (pump–probe geometry) in which both pump pulses are collinear and the probe pulse intersects at the sample with a small crossing angle (less than 10°). Briefly, instead of a single pump pulse yielding two simultaneous optical field interactions followed by a probe field, as in TA spectroscopy, 2DES utilizes two pump pulses separated in time by *t*_1_, known as the coherence time, followed by a third probe pulse after *t*_2_, known as the waiting time. The third-order nonlinear polarization generated by the probe pulse emits a field which is self-heterodyned with the probe pulse in a partially collinear pump–probe geometry, resulting in the measurement of 2D absorptive spectra.^17^ The phase-locked pump pulses were generated using the Translating-Wedge-Based Identical-Pulses-eNcoding System (TWINS) technology, as described elsewhere.^17−19^ The CPA system is the same as in the TA experiments; however, a different NOPA was used to achieve high spectral bandwidth and simultaneous high time resolution (sub 20 fs) after compression using multiple bounces on a pair of double-chirped mirrors (DCM). The pulses generated in the NOPA were split at an inconel-type beam splitter into the pump and probe paths; the pump path is sent through the TWINS system and subsequently recompressed using another pair of DCMs. A 100 μm piece of glass is placed in the probe path to reduce chirp, as measured by a polarization-gated cross-correlation between the pump and probe pulses at the sample position. To acquire 2D spectra, the delay between the two pump pulses is rapidly scanned with interferometric stability in the TWINS. The Δ*T*/*T* spectrum at each fixed pump1–pump2 and pump2–probe delay time is measured. Simultaneously, the pump1–pump2 interferogram is measured on a photodiode, which is used for proper phasing of the Fourier-transformed spectrum in postprocessing. This rapid scanning process proceeds at each pump2–probe delay time, from −1 to 60 ps maximum; coherence analysis is performed on scans from −500 fs to 2 ps. The samples were contained in a quartz cuvette with 200 μm path length.

### Data Analysis

All data analysis was performed in Matlab 2021. Global fitting of both TA and 2DES data was performed with in-house developed code.

### Density Functional Theory

Geometry optimizations of the oligomer structures (from monomer to trimer), graphene nanoflakes, and the different interfaces have been performed at the DFT level of theory, considering the long-range corrected wB97xD functional^20^ and the 6-31G(d,p) basis set within the Gaussian16 software.^21^ From the optimized structures, single point time-dependent DFT (TD-DFT) calculations were performed to obtain the absorption spectra.

The work function (WF) analysis has been carried on a model interface consisting of a monomer unit chemically bonded to a graphene supercell. Periodic boundary conditions were used to simulate a full monolayer, in which the *c* direction was considered as the normal to the graphene surface. The supercell dimensions were set at *a* = 10.70 Å, *b* = 6.179 Å, *c* = 40 Å, α = 120^*r*^*c*, β = 90^*r*^*c*. Vector *c* was set to 40 Å to create a vacuum area above the interface to avoid interactions between the repeating units. The interfaces were analyzed with the Quantum Espresso 6.5 suite of programs.^22^ Ultrasoft pseudopotentials,^23^ together with PBE functional and with 50 and 330 Ry cut-offs for wave functions, and charge density, respectively, were used. Dipole corrections were applied to maintain a constant, zero external electric field along the normal to the interface

## Results and Discussion

### Steady-State Absorption, PL, and Photocurrent

The optical properties of Ph_2_TDPP, EXG–TDPP, and c-EXG–TDPP were first investigated by measuring the linear absorption and steady-state PL of toluene-dissolved solutions (Figure 1b). For Ph_2_TDPP solutions, two main absorption bands are present.^16^ The dominant band ranges from 1.97 to 2.48 eV (500–630 nm), including two strong and narrow features at 2.063 eV (601 nm) and 2.202 eV (563 nm) attributed to the vibronic progression of intramolecular CT (intra-CT) states associated with the electron-donating thiophene rings and the strongly electron-accepting diketopyrrolopyrrole (DPP) moiety,^24−26^ although the intra-CT nature of this system has been questioned.^27^ Monomeric Ph_2_TDPP features strong PL with a vibronic progression showing PL lines at 2.0, 1.83, and 1.67 eV, which is the mirror image of the vibronic progression in linear absorption, suggesting a small electronic reorganization energy.^28,29^ The non-cross-linked nanohybrid system, EXG–TDPP, exhibits absorption that is red-shifted compared to Ph_2_TDPP by 55 meV (15.2 nm) with a slight broadening, and a PL spectrum with a similar vibronic progression structure as in Ph_2_TDPP (Figures S1 and S2, Supporting Information (SI)). The integrated PL is reduced by a factor of 4.3 compared to Ph_2_TDPP. Strikingly, the cross-linked c-EXG–TDPP nanohybrid shows a 224 meV (67.4 nm) red-shift in absorption compared to the monomer (Figure 1) and is significantly broadened compared to both Ph_2_TDPP and EXG–TDPP. The PL intensity is reduced by a factor of 147 compared to Ph_2_TDPP. We sometimes refer to the c-EXG–TDPP nanohybrid as “blue graphene”, due to its visible blue color compared to the pink observed for Ph_2_TDPP.^16^

The red-shifts and quenching observed here in increasing strength from EXG–TDPP to c-EXG–TDPP are likely explained by a few factors. As reported previously,^16^ the electronic properties of both the EXG and Ph_2_TDPP/PhTDPP (modified monomer in EXG–TDPP^16^) are modified by covalent bonding in the nanohybrid. This likely explains the 10–20 nm red-shift in EXG–TDPP. Additionally, significant oligomerization occurs in the cross-linked c-EXG–TDPP, which can lead to an increase in conjugation length, and subsequent red-shift and broadening due to conformational disorder.^30−33^ We have also confirmed the presence of a CT process in c-EXG–TDPP thin films using steady-state photocurrent measurements (SI section 12). Although it is well-known that solvents play a large role in modulating the efficiency of CT processes,^28,34−36^ thus making a direct comparison between solid state and solution systems difficult, the measurable photocurrent in the solid-state system provides some evidence that the CT process may also occur in solution. We further confirm this CT process via TA and 2DES and show that the CT states contribute to the significant red-shift observed in c-EXG–TDPP relative to Ph_2_TDPP.

### Ultrafast Transient Absorption of Ph_2_TDPP, EXG–TDPP, and c-EXG–TDPP

To understand the energy and charge transfer processes resulting in PL quenching and the nature of the dramatically red-shifted linear absorption in c-EXG–TDPP compared to monomeric Ph_2_TDPP, we performed TA spectroscopy out to 1 ns pump–probe delay time (Methods).

**Figure 2:**
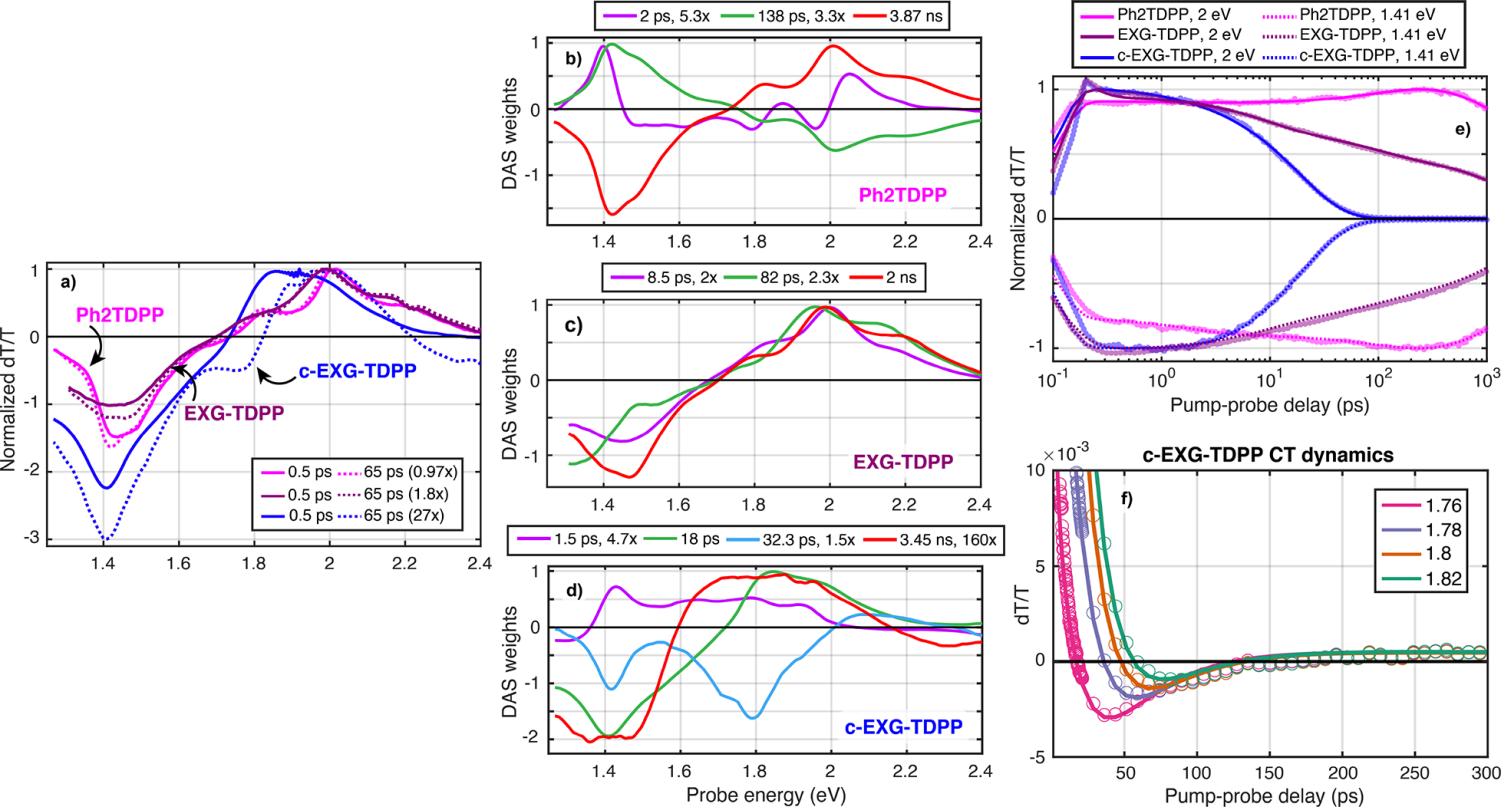
Transient absorption and global fitting of Ph_2_TDPP, EXG–TDPP, and c-EXG–TDPP. (a) Comparison of Ph_2_TDPP (pink), EXG–TDPP (purple), and c-EXG–TDPP (blue) Δ*T*/*T* signals at 0.5 ps (solid line) and 65 ps (dashed line). The signals have been multiplied by the factors indicated in the legend for easy comparison between data sets. The Ph_2_TDPP data set is pumped at 575 nm with 2.3 μW average power, EXG–TDPP is pumped at 565 nm with 70 μW average power, and the c-EXG–TDPP is pumped at 640 nm with 20 μW average power, yielding comparable strength Δ*T*/*T* signals at early times. (b, c, d) DAS for Ph_2_TDPP, EXG–TDPP, and c-EXG–TDPP, respectively. (e) Δ*T*/*T* dynamics at energies 1.41 eV (dashed), and 2 eV (solid), with data points indicated by translucent symbols and global analysis fits as solid lines. Each trace is normalized by its maximal absolute value. (f) Charge transfer dynamics in c-EXG–TDPP, with probe energies labeled in the legend. Solid lines are global fit results and empty circles are data points.

Monomer TA studies were performed on Ph_2_TDPP dissolved in toluene (Figure 2a, pink curves). Three types of features are observed in differential transmission (Δ*T*/*T*) spectra: (1) positive ground state bleaching (GSB) due to depletion of the ground state coincident with vibronic progression features at 2.2 and 2.06 eV for the S_0_ → S_1_ transitions, (2) positive stimulated emission (SE) features resulting from amplification of the probe and coincident in energy with the steady-state PL features at 2.0, 1.83, and 1.68 eV, and (3) negative excited-state absorption (ESA) features due to photoinduced absorption of the probe on S_1_ → S_*n*_ transitions around 1.42–1.49 eV. Aside from small shifts in the SE peak energies and ESA, as well as an anisotropy decay that will be discussed later, the TA signal dynamics at 1.41 (ESA), 1.8 eV (ESA/GSB), and 2 eV (GSB/SE) do not decay much within the 1 ns pump–probe delay range. These features, especially the comparably strong GSB and SE bands, are in agreement with the high PL quantum yield observed in DPP-derivatives.^16^

In stark contrast, the TA spectra and dynamics for the cross-linked c-EXG–TDPP polymer system in toluene (Figure 2a, blue curves) differ significantly from Ph_2_TDPP. The positive features observed between 1.7 and 2.3 eV for c-EXG–TDPP, which are coincident with linear absorption, are attributed to GSB; the SE band may overlap either with the GSB or ESA features, as evidenced by the weak but overlapping steady-state PL (Figure 1). The GSB features are broadened compared to monomeric Ph_2_TDPP and red-shifted by around 230 meV. Negative ESA features extending from 1.7 eV well-beyond 1.3 eV (Figure S4) consist of a narrow peak at around 1.42 eV resembling the ESA features in Ph_2_TDPP superimposed on a very broad feature with line width greater than 300 meV. The presence of the narrow ESA feature indicates that c-EXG–TDPP partially retains a similar electronic structure in the excited state as in Ph_2_TDPP, while the dynamics of this feature follow the fast 18 ps quenching dynamics of the broad GSB features. At a pump–probe delay time of 65 ps (Figure 2a, blue dashed curve), a new negative photoinduced absorption (PIA) signal is observed around 1.8 eV (note that the TA signal in Figure 2 at 65 ps for c-EXG–TDPP is magnified by a factor of 27). This PIA signal, which is discussed in further detail later in the article, is attributed to charge transfer (CT)^37^ in the nanohybrid system. The c-EXG–TDPP TA signal decays almost completely within 100 ps at all probe energies. These findings are consistent with the PL quenching observed in the c-EXG–TDPP nanohybrid, seen here as the decay of the GSB and potentially SE bands, which takes place on the time scale of tens of picoseconds. These dynamics are examined quantitatively in the following global analysis.

To consistently extract lifetimes and spectra from the Ph_2_TDPP, EXG–TDPP, and c-EXG–TDPP TA experiments, we analyzed the data using global fitting. In this procedure, a fixed number of exponential decays are chosen to simultaneously fit the TA spectra at all pump–probe delay times, where only the spectral weights, global lifetimes, and probe wavelength-dependent pump–probe overlap times (chirp) are allowed to vary during the fitting procedure.^38^ The resulting spectra are referred to as decay-associated spectra (DAS) (SI section 11). The global fitting procedure provides more consistent lifetimes than fitting at a single wavelength in the TA spectrum and can provide immediate insights into the physical processes taking place in the system such as energy and charge transfer, as well as structural transformations that may manifest as spectral shifts. For the TA experiments, either three (Ph_2_TDPP) or four (EXG–TDPP, c-EXG–TDPP) exponential decays are used to fit the TA spectra.

#### Global Fitting of Ph_2_TDPP TA

In the case of Ph_2_TDPP, a minimum of three lifetimes are necessary to properly fit the spectra. We extracted lifetimes of 2 ps, 138 ps, and 3.87 ns. The longest lifetime, 3.87 ns (chosen as a fixed parameter in the fitting procedure with accuracy limited by the 1 ns measurement window),^16^ can clearly be associated with radiative recombination and is dominant in strength (3.3× times larger than the 138 ps DAS at maximum signal); the two GSB, three SE features, and ESA features are all captured in this DAS.

The second most prominent DAS with lifetime of 138 ps is almost exactly the negative attenuated mirror image of the radiative relaxation DAS (factor of 3.3× smaller in Figure 2b). This DAS captures the slow rise of the GSB dynamics at 2 eV (Figure 2e, pink curve) and the increase in ESA at 1.41 eV. We confirm via time-dependent TA anisotropy measurements (Figure S11) that this DAS reports on rotational diffusion of Ph_2_TDPP in toluene.^39−42^ Note that the TA experiments reported here, unless otherwise stated, were performed with cross-polarized pump and probe, to reduce the effects of pump scattering. The Ph_2_TDPP anisotropy was fit at 2.01 eV (overlap of GSB and SE bands) and shown to decay with a lifetime of 170.2 ± 15.2 ps, in good agreement within error bars with the 138 ps lifetime extracted with global fitting (Figure S11).

The third DAS, with a lifetime of approximately 2 ps, is intriguing, as it indicates that the states associated with SE at 2.0, 1.83, and 1.68 eV relax to lower energies. This relaxation is revealed by the dispersive DAS, with negative (positive) Δ*T*/*T* features on the red (blue) side of the transition, indicating that the SE transitions red-shift after 2 ps. This relaxation is also correlated with an increase in the ESA at 1.4 eV, implying that this shift takes place on the excited-state potential energy surface. We provide two possible explanations, which will be explored in more detail: that the red-shift is caused by either intramolecular vibrational redistribution (IVR) in the excited state^27,43−50^ or conformational changes related to torsional motion of the electron-donating phenyl-thiophene unit bound to the DPP moiety, which may be related to the intra-CT character of Ph_2_TDPP.^51,52^

#### Global Fitting of c-EXG–TDPP TA

We also performed global fitting analysis of cross-linked c-EXG–TDPP TA spectra (Figure 2d). In this sample, four exponentials were required to adequately fit the data; the resulting fit yields lifetimes of 1.5 ps, 18 ps, 32.3 ps, and 3.45 ns (fixed parameter). The fastest DAS with a lifetime of 1.5 ps shows GSB decay of a broad distribution of states that are red-shifted from the main linear absorption features, ESA at energies lower than 1.36 eV, as well as a positive feature coincident with ESA in the 18 ps DAS. This last feature can be interpreted as the in-filling of excited states, seen as an increase in ESA within 1.5 ps. The GSB decay features may correspond to decay of conformational subunits^51^ of longer conjugation length Ph_2_TDPP states, which are expected to be red-shifted from shorter conjugation subunits.^30,32^ The oligomer may be broken into subunits via twisting between TDPP units^51^ or homocoupling defect sites.^53,54^ Within 1.5 ps, these states may decay due to energy transfer either to EXG or to the TDPP-EXG covalent bond site; we are unable to determine whether this process takes place in parallel or sequentially with the 18 ps process. We also do not rule out the possibility that an SE band overlaps with the positive features for the 1.5 ps DAS; indeed, the measured steady-state PL (Figure 1) of c-EXG–TDPP shows very weak and broad PL slightly red-shifted from the linear absorption.

The dominant DAS with a lifetime of 18 ps resembles the TA spectra at early times, with GSB coincident with linear absorption, and two overlapping ESA features centered around 1.4 eV: a narrow feature associated with the TDPP unit and a much broader feature likely associated with covalently bound EXG. The main GSB features are red-shifted relative to Ph_2_TDPP due to hybridization of the electron-donating phenyl-thiophene unit with EXG. This hybridization may be evident by the two-component ESA feature, with a narrow feature that resembles ESA of Ph_2_TDPP, preserving some excited state character from the dye unit, while a new broad feature appears that may be correlated with delocalized states at the sp^3^ defect in EXG.

Significantly, a new PIA feature (negative feature at 1.8 eV, see Figure 2f) is observed in the DAS with a lifetime of 32 ps, which is likely indicative of the CT product state. This DAS also contains the Ph_2_TDPP-associated ESA feature at 1.42 eV and weaker GSB around 2.1 eV. The spectral features of the PIA related to the CT state will be investigated further in the 2DES section. After 32 ps, these features decay, and the signal at all probe energies nearly reaches zero aside from the 3.45 ns DAS, which is at least 160 times smaller in strength than the 18 ps DAS. Thus, the CT state either further separates into a dark charge-separated state distributed throughout the cross-linked c-EXG–TDPP scaffold, generating steady-state photocurrent (SI. section 12), or recombines nonradiatively via internal conversion. The slowest and weakest DAS with the 3.45 ns lifetime shows very broad GSB from 1.6 to 2.2 eV and broadened ESA features, indicating the presence of many disordered and nearly dark states.

#### Global Fitting of EXG–TDPP TA

Additionally, TA experiments were performed on the model system EXG–TDPP (Figure 2c), which consists of monomeric PhTDPP units covalently bound to EXG on both sides of individual nanosheets (Figure 1); no cross-linking of different EXG flakes occurs in this nanohybrid.^16^ The TA spectra and dynamics more closely resemble that of Ph_2_TDPP (Figure S12): in contrast to c-EXG–TDPP, a strong SE band is apparent, in agreement with the small PL quenching factor of around 4 compared to Ph_2_TDPP. Four exponentials were necessary to adequately fit the spectra, resulting in lifetimes of 65 fs, 8.5 ps, 82 ps, and 2 ns (see Figure S12 for fast DAS). The fastest DAS is associated with the coherent artifact during pump–probe pulse overlap. No obvious CT state features that might be associated with new negative peaks are observed in this nanohybrid; in fact, the 8.5 ps and 2 ns DAS are quite similar to each other, aside from increased ESA in the 2 ns DAS. The 82 ps DAS shows slightly red-shifted GSB and SE features around 1.9 eV, as well as a red-shifted ESA feature around 1.3 eV. This red-shift might be attributed to energy transfer by Dexter or Förster processes.^7,14,55^ The 8.5 and 82 ps lifetimes may be attributed to charge/energy transfer between PhTDPP units and subsequent back electron transfer, which eventually results in radiative recombination with a lifetime of 2 ns. One important point is that both EXG–TDPP and c-EXG–TDPP show ESA signals which are much broader and extend deeper into the NIR than Ph_2_TDPP (SI section 9). Because these features are common to both systems that involve sp^3^ defects at the covalent bond site, the broad ESA is attributed to an EXG defect, in agreement with literature reports of EXG spectral features in this energy range.^55^ This comparison of TA spectra between the cross-linked c-EXG–TDPP and not-cross-linked EXG–TDPP nanohybrid indicates that charge transfer, rather than energy transfer, between different EXG flakes is the process that leads to strong PL quenching in c-EXG–TDPP.

### Ultrafast Two-Dimensional Electronic Spectroscopy and Global Analysis

To further investigate the transient optical properties of both Ph_2_TDPP and c-EXG–TDPP, we performed ultrafast 2DES, which is a third-order nonlinear spectroscopy method similar to TA but with the addition of a second pump pulse at a scanned time delay *t*_1_. For a given waiting time between the second pump and the probe, *t*_2_, scanning *t*_1_ yields the excitation frequency axis via Fourier transform and allows for simultaneously high temporal and frequency resolution.^56−58^ The 2DES spectrometer^17,18^ and experimental setup are discussed in Methods. The simultaneously high spectral and time resolution provided by 2DES allows for the observation of phenomena including vibrational relaxation,^59,60^ intramolecular vibrational redistribution,^48^ charge and energy transfer,^61^ as well as coherent oscillations that reveal vibrational state splitting in both the excited and ground states.^50,60^ Significantly for the systems under study here, 2DES allows us to better understand how the pump photon energy affects relaxation processes such as energy and charge transfer.

**Figure 3:**
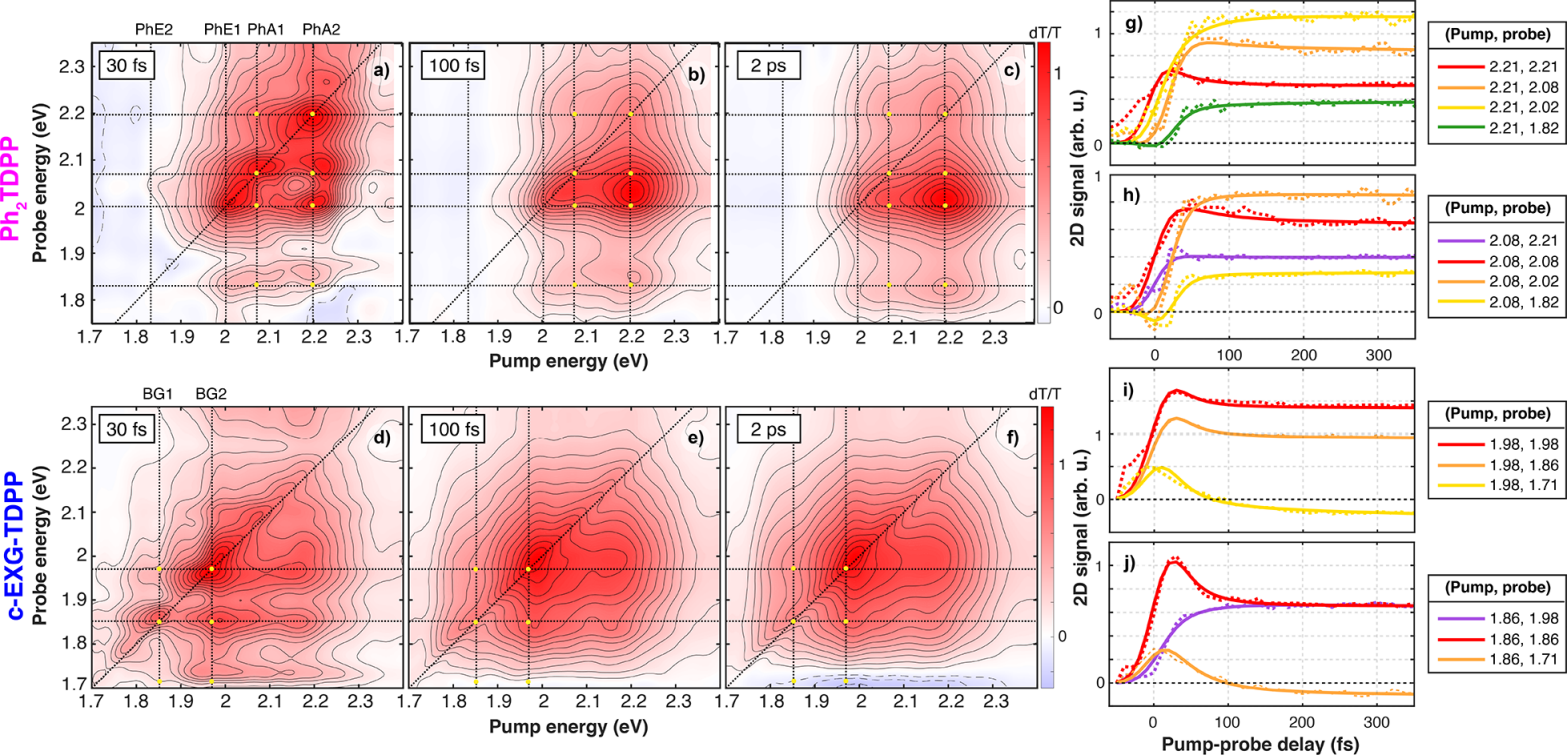
Selected 2D absorptive spectra for Ph_2_TDPP and c-EXG–TDPP. (a–f) 2D absorptive maps at 30 (a, d), 100 (b, e), 2000 (c, f) fs for Ph_2_TDPP (a–c) and c-EXG–TDPP (d–f), with color bar shown in top right (blue for negative, zero is set to white, red for positive Δ*T*/*T*). Points of interest are indicated by yellow dots in all maps, which coincide with pumping the linear absorption of Ph_2_TDPP (PhA_1_, PhA_2_), probing the PL of Ph_2_TDPP (PhE_1_, PhE_2_), or pumping/probing the c-EXG–TDPP linear absorption (BG1, BG2) and also probing the c-EXG–TDPP ESA. The corresponding dynamics at those points are shown in panels g–j (g, h for Ph_2_TDPP, i, j for c-EXG–TDPP). Solid (dashed) contour lines correspond to positive (negative) 2D signals.

Selected 2DES maps at waiting times *t*_2_ = 30 fs, 100 fs, and 2 ps for both Ph_2_TDPP and c-EXG–TDPP are shown in Figure 3. 2DES on EXG–TDPP was not performed due to deleteriously high scattering. Dynamics (dashed lines) and global fit results (solid lines) from −100 to 350 fs are shown at selected pump and probe energies (*E*_pump_ = *ℏω*_1_, *E*_probe_ = *ℏω*_3_) points (yellow dots in Figure 3g–j). The instrument response function (IRF), corresponding to the temporal resolution of the experiment, is estimated to be between 15 and 22 fs, as measured using a polarization-gated cross-correlation and fitting of the rise-time of the signal build-up along the diagonal in the 2DES experiments.

For the Ph_2_TDPP 2DES maps, at least four features are evident in the energy ranges from 1.7 to 2.4 eV: two GSB features labeled PhA_1_ and PhA_2_, and two SE features labeled PhE_1_ and PhE_2_ (dashed lines in Figure 3a–c). These four features, PhA_1_/PhA_2_ and PhE_1_/PhE_2_, are coincident with the vibronic progression peaks observed in linear absorption and PL. The rise-times of the diagonal features at PhA_1_ and PhA_2_ are limited by the IRF. Within *t*_2_ = 30–40 fs, substantial cross-peaks develop (Figure 3a) including the above-diagonal peak between PhA_1_ and PhA_2_, indicating either strong coupling or very rapid mixing of the two vibronic states possibly due to excitonic self-localization.^62−67^ Excitonic self-localization is the subpicosecond process through which optical excitation with excess photon energy generates mobile excitons which then self-trap via local distortions of the underlying lattice.^66^ This process has been shown to reduce the early time anisotropy below the expected 0.4 for parallel dipoles in randomly oriented solution.^64^ We measured an early time anisotropy value of 0.17 in Ph_2_TDPP, providing further evidence for self-localization (SI section 11).

In addition to the above-diagonal cross-peak between the two PhA_*n*_ transitions, we see many cross-peaks below the diagonal between the two PhA_*n*_ transitions and each PhA_*n*_ transition and the PhE_*n*_ transitions. The below-diagonal peaks between PhA_*n*_ and PhE_*n*_ feature a slower rise and delayed onset on a time scale of around 45 fs; thus, SE is established very rapidly, indicating ultrafast partial relaxation of the excited electronic states (see SI section 6 for 2DES time resolution discussion). Over the course of 2 ps, the below-diagonal (*ℏω*_1_, *ℏω*_3_) = (PhA_1_, PhE_1_) and (PhA_1_, PhE_2_) features relax to the energies measured in the steady-state linear absorption and PL (Figure S2).

The 2DES maps of the cross-linked c-EXG–TDPP nanohybrid (Figure 3d–f) show evidence of strong coupling between the states observed in linear absorption, labeled here BG1 and BG2 (blue-graphene 1 and 2). This approximately 45 fs relaxation process, as determined by global fitting (next section, also S.5), is quite prominent when exciting at BG2 and probing at BG2, BG1, and the ESA band. In this case, the diagonal signal drops by around 40%, while the above-diagonal feature at (*ℏω*_1_, *ℏω*_3_) = (BG1, BG2) and lower energy ESA signal form on the same time scale. This rapid relaxation process is again potentially attributed to excitonic self-localization. Additionally, even at very early times, a signal is observed for both pump and probe energies as high as 2.3 eV, with prominent below-diagonal features at (*ℏω*_1_, *ℏω*_3_) = (2.16, 1.98) eV and (2.16, 1.85) eV. These early time below-diagonal GSB signals indicate either rapid energy transfer or a shared ground state resulting from strong coupling; the nature of the features located at higher energies will be discussed in the following 2D global analysis section.

#### Global Analysis of Ph_2_TDPP and c-EXG–TDPP 2DES

**Figure 4:**
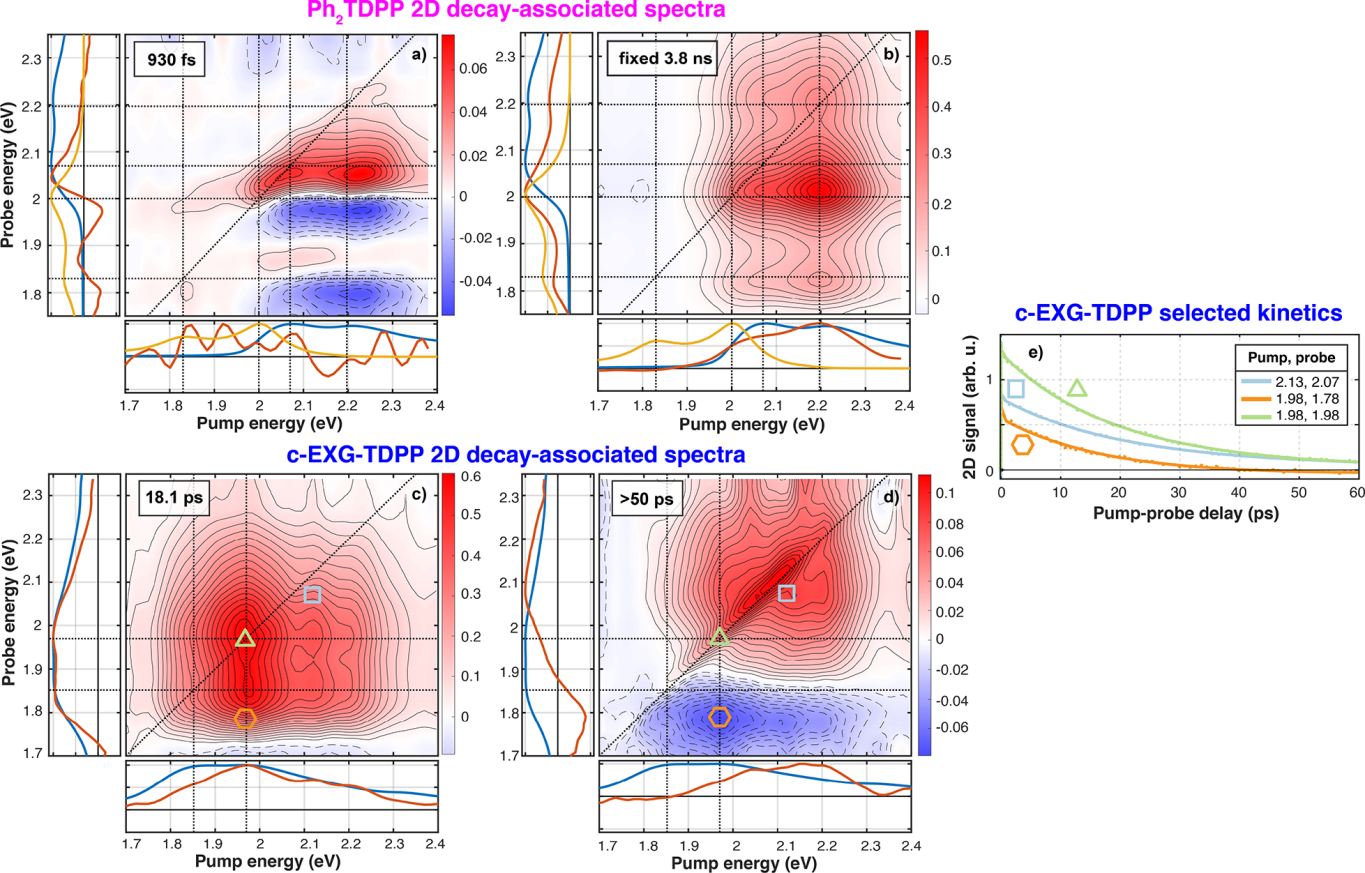
2D decay-associated spectra for Ph_2_TDPP (a, b) and c-EXG–TDPP (c, d), and selected dynamics for c-EXG–TDPP (e). The lifetime of each DAS is shown in the top-left corner of each 2D-DAS map; the two most physically significant 2D-DAS are shown here, the two remaining 2D-DAS for each sample as shown in SI section 5. Subplots are also shown along the pump and probe energy axes; blue is the linear absorption of the sample, yellow is the steady-state PL (Figure 1), and red are the sums along the probe and pump energies. The 2D dynamics are also shown for selected pump/probe energies for c-EXG–TDPP, with corresponding symbols in the 2D 2D-DAS; a negative Δ*T*/*T* signal is observed when pumping at 1.98 eV and probing at 1.78 eV. Contour spacing is equal to the maximum positive value of the color bar divided by 20. Solid (dashed) contour lines correspond to positive (negative) 2D signals.

Clear insight into these processes is provided by global analysis of the 2DES absorptive spectra resulting in two-dimensional DAS (2D-DAS) (see SI section 11 for details on 2D global analysis fitting). For the 2DES experiments, four 2D-DAS were used to fit both Ph_2_TDPP and c-EXG–TDPP data sets. An additional DAS is required for the 2DES experiments compared to TA due to the much higher time resolution, allowing us to resolve faster distinct physical processes. The two most physically significant 2D-DAS are shown in Figure 4; all four 2D-DAS for both samples are shown in SI section 5.

In the case of Ph_2_TDPP, the four lifetimes resulting from the 2DES global fitting are 13 fs, 45 fs, 930 fs, and 3.8 ns (fixed parameter). The fastest 2D-DAS is associated with the coherent artifact because its duration is on the same time scale as the IRF (approximately 22 fs). The next fastest 2D-DAS (45 fs) shows decay (positive 2D-DAS) of both the PhA_1_/PhA_2_ features on the diagonal, as well as negative below-diagonal cross-peaks, coinciding with an in-filling of the SE features at 2 and 1.8 eV (Figure S7). This 2D-DAS confirms that some relaxation takes place in the excited state leading to SE within 45 fs, extremely rapidly compared to the radiative relaxation time of 3.8 ns. The next two 2D-DAS (930 fs and 3.8 ns) are shown in Figure 4a,b. The 3.8 ns 2D-DAS shows a clear eight-peak structure, with diagonal and cross-peak features associated with PhA_1_ and PhA_2_ and below-diagonal features indicating SE at PhE_1_ and PhE_2_ when pumping PhA_1_ and/or PhA_2_. When the 2D-DAS is integrated over the pump (probe) energies, the resulting spectra clearly match the measured PL and linear absorption spectra, respectively (Figure 4b red, yellow, and blue curves in insets), indicating that the system has reached a quasi-equilibrium on these time scales.

The 930 fs 2D-DAS (Figure 4a) shows relaxation indicated by a dispersive line shape located at the SE signals along the probe energy, where there is a decay on the high energy side and in-filling of the low energy side. This relaxation is similar to the features observed in the TA experiments (Figure 2b). What was not possible to resolve in the TA but is made clear in the 2DES experiments is that the red-shift is only evident in the SE features upon pumping the linear absorption resonances of PhA_1_ and PhA_2_. Strikingly, the nodes (zero-crossings) of this 2D-DAS as a function of probe energy occur at almost exactly the energies of the steady-state PL features (PhE_1_ and PhE_2_). No discernible energy shifts of the diagonal GSB features are observed, given the decaying components along the diagonal. With this 930 fs 2D-DAS, 2DES spectroscopy allows us to resolve relaxation in the excited state,^49,50^ which was not immediately evident from the single-color TA measurements. This relaxation is consistent with either IVR^49^ or torsional relaxation common to conjugated polymers.^51,52^

We begin by exploring the first possibility of IVR. It is well-known that broadband excitation can generate coherent wavepackets that lead to measurable oscillations, imparting information about both the excited- and ground-state potentials.^48,60,68^ One dephasing mechanism of these oscillations is IVR that manifests as rapid vibrational state population decay. Prior to IVR dephasing, typically observed to take place within a few picoseconds,^44,45,49^ the excited state SE signal is effectively blue-shifted, which may explain the relaxation DAS observed in Ph_2_TDPP. In contrast, strong and long-lived oscillations are observed in the 2DES experiments for both Ph_2_TDPP and c-EXG–TDPP (Figures S14 and S15), with dephasing lifetimes longer than the 930 fs 2D-DAS lifetime reported here. However, it is difficult at this time to disentangle the role of toluene as the solvent with its potential vibrational spectator modes and to ascertain if we are directly detecting IVR in the Ph_2_TDPP system.

Turning now to the second possible explanation of the observed relaxation, we consider an ultrafast conformational change. In particular, we consider a torsional change between the DPP moiety and phenyl-thiophene unit. In conjugated polymers, it is often reported that the ground state is more twisted than the excited state.^31,33,51,52^ Thus, a dynamic planarization is expected to occur in the excited state upon electronic excitation. For example, in a donor−π-acceptor system in which the acceptor molecule is a DPP-derivative,^52^ torsional motion of the thiophene unit relative to the DPP moiety was shown to occur within 400 fs, which is a comparable time scale to the relaxation DAS lifetime observed in Ph_2_TDPP. However, our analysis of oscillatory signals in both Ph_2_TDPP and c-EXG–TDPP (SI section 10) shows that vibrational coherent wavepacket motion persists after photoexcitation for more than 1 ps, and the dephasing due to population decay caused by IVR likely occurs on time scales longer than 1 ps. Thus, torsional relaxation is a more acceptable explanation for the relaxation DAS observed in this system.

The 2D global analysis technique was then applied to the cross-linked c-EXG–TDPP system. Two separate analyses were performed, one analysis on the short waiting time scans (−100 to 2000 fs with 10 fs steps) and another on the longer waiting time scans (−100 fs to 60 ps). The longer time scale 2D-DAS are shown in Figure 4, while the remaining 2D-DAS are shown in SI section 5. The following lifetimes are recovered in the global analysis in the two separate analyses: 15 fs, 42 fs, 355 fs, 18.1 ps, and >50 ps, with the accuracy of the latter limited by the maximum scanned *t*_2_ delay in the 2DES data of 60 ps. As in the Ph_2_TDPP system, the fastest 2D-DAS (15 fs) is associated with the coherent artifact. The 45 fs 2D-DAS shows above-diagonal in-filling (negative 2D-DAS) when pumping the lower energy BG1 transition and probing the higher energy BG2 transition. On the same time scale, decay of GSB is observed when probing below-diagonal and pumping between 1.8 and 2.3 eV, into the high energy tail of the linear absorption features, as was seen in the early time dynamics (Figure 3i,j). This fast 2D-DAS is again attributed to excitonic self-localization^62−67^ which apparently does not dephase coherent oscillations (SI section 10 for coherent oscillations in c-EXG–TDPP). The last subpicosecond 2D-DAS (355 fs) bears a strong resemblance to the 1.5 ps DAS observed in TA when using narrowband pumping at 1.938 eV, showing the decay of GSB features at probe energies smaller than 2 eV. Global fitting of the 2DES data shows here that these states, which are attributed to conformational subunits of the TDPP oligomeric bridge between EXGs, have already been populated before 355 fs, with the below-diagonal GSB decaying on this time scale. The discrepancy in time scales between global fitting of the TA (1.5 ps) and 2DES (355 fs) is not yet understood but could arise potentially due to differences in pump fluence, narrowband versus broadband excitation, and the difference in pump–probe delay sampling points between the TA and 2DES experiments.

One notable difference between Ph_2_TDPP and c-EXG–TDPP is the 930 fs excited state relaxation observed in the former. First, it is noted that the broad PL in c-EXG–TDPP overlaps with and is slightly red-shifted from the linear absorption (Figure 1). In this spectral region, no such relaxation DAS associated with a red-shift is observed in c-EXG–TDPP. Thus, if the torsional relaxation explanation is accepted for Ph_2_TDPP, one of two statements about c-EXG–TDPP is possible: (1) that torsional relaxation does not occur in c-EXG–TDPP, and (2) that the relaxation is not observable due to weak SE or overlap with ESA, and thus no probe of the excited electronic state properties is available. At this time, it is not possible to definitely claim here that torsional relaxation does not occur in c-EXG–TDPP, but it is probable. Previous reports of dynamic Stokes shifts in conjugated polymers^33^ showed that the torsional relaxation leading to the shift occurred in solution but not in film due to lack of torsional freedom in the solid state. A similar situation may arise in the disordered c-EXG–TDPP composite system that may possess increased rigidity along the oligomeric TDPP backbone confined in between EXG nanosheets.

The longer 2D-DAS, which are determined by global fitting the 2DES data from −100 fs to 60 ps, have lifetimes of 18 ps and approximately 50 ps (Figure 4c,d). The 18 ps lifetime 2D-DAS is in agreement with the lifetime of the dominant DAS in TA. In the 2D-DAS, the 18 ps component corresponds to strong GSB at the high energy BG2 transition, the below-diagonal GSB (and potentially SE) cross-peak between BG2 and BG1, weaker GSB on the diagonal at BG1, and a signal at pump/probe energies higher than 2.05 eV. The states associated with this 2D-DAS absorb most strongly at the BG2 resonance and higher energies (Figure 4c, bottom panel), with the signal found at probe energies with the same spectrum as in linear absorption (Figure 4c, left panel). We do not rule out the possibility of SE, as seen in this 2D-DAS, because the system absorbs strongly at BG2 and emits at BG1.

The last 2D-DAS, with a lifetime close to 50 ps (the uncertainty on lifetime determination is high because the waiting time was only scanned to 60 ps), provides the clearest evidence for a photoinduced CT state in c-EXG–TDPP (Figure 4d). When the higher energy linear absorption resonance BG2 at 1.98 eV is pumped, a new PIA feature appears centered at 1.78 eV below the diagonal. This feature is confirmed to be PIA by examining the long time dynamics (Figure 4e, orange curve, hexagon symbol on 2D map; Figure 2f): furthermore, the TA signal becomes negative after 45–50 ps. This 2D-DAS also captures GSB of a higher energy subset of states lying between 1.95 and 2.3 eV; this subset could correspond to lower conjugation length subunits of the oligomeric backbone chain, such as the units directly bound to EXG, or conformational subunits. Integrating along the pump energy axis, a broad PIA band is evident, which agrees with the 32 ps DAS observed in TA when pumping at 1.938 eV. Significantly, minimal PIA is observed when pumping the BG1 resonance, indicating that the electronic reorganization required for charge transfer takes place only when pumping at energies higher than approximately 1.9 eV. It is unlikely that the negative feature is ESA associated with the new states observed via GSB from 1.95 to 2.2 eV, because the pump energy dependence is quite different for the two signals: the proposed PIA signal is strongest when pumped in a narrow band around 1.98 eV, whereas the GSB of the higher lying states spans the pump energy range from 1.85 to 2.3 eV. The narrow feature along the diagonal from 1.95 to 2.2 eV is likely due to pump–pump scatter, rather than inhomogeneous broadening.^69,70^

Through the use of global fitting, the relatively featureless 2DES spectrum for c-EXG–TDPP (Figure 3) is cleanly separated into constituent spectra with different lifetimes (Figure 4), enabling advanced interpretation of the electronic dynamics. The implementation of 2DES has additionally provided crucial information about the pump energy dependence of the formation of the CT state at 1.78–1.8 eV that would otherwise be difficult to obtain via TA.

### Density Functional Theory Calculations

**Figure 5:**
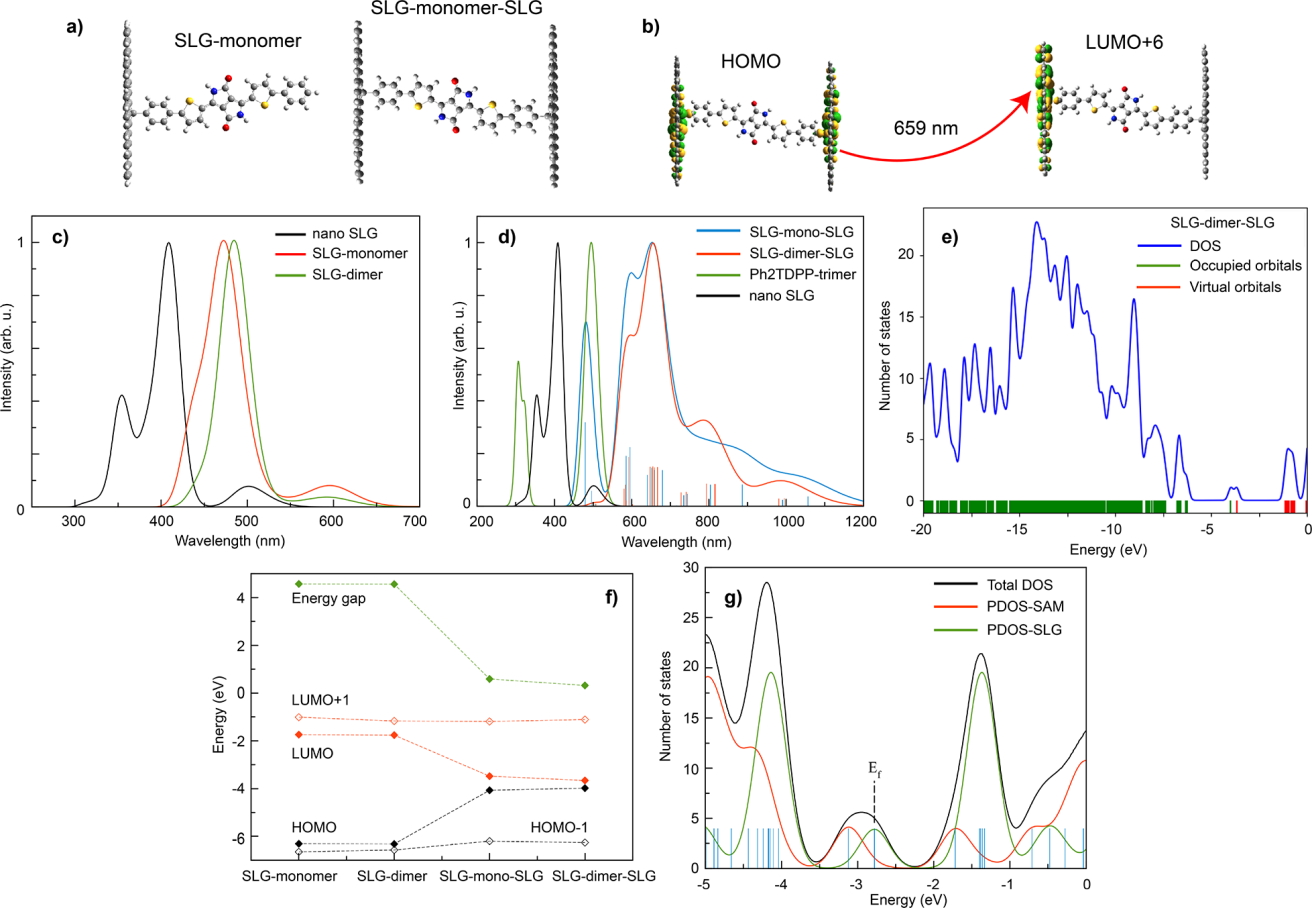
DFT calculation results. (a) Chemical structure of SLG–monomer and SLG–monomer–SLG systems used in calculations. (b) Example transition at 659 nm (1.882 eV) between HOMO and LUMO+6 levels in SLG–monomer–SLG system showing photoinduced charge transfer between TDPP-graphene covalent bond sites. (c) Calculated linear absorption spectra of nano-SLG (black), SLG–monomer (red), and SLG–dimer (green) systems. (d) Calculated linear absorption spectra of nano-SLG (black), Ph_2_TDPP-trimer (green), SLG–monomer–SLG (blue), and SLG–dimer–SLG (red). (e) Density of states (DOS) for the SLG–dimer–SLG system, with occupied (green) and unoccupied (red) orbitals indicated at bottom of figure. (f) Band-gap, HOMO–1, HOMO, LUMO, and LUMO+1 energies for SLG–monomer, SLG–dimer, SLG–mono–SLG, and SLG–dimer–SLG. (g) Projected density of states for all three systems, where SAM refers to a self-assembled monolayer of Ph_2_TDPP molecules.

Density functional theory (DFT) calculations were performed to simulate the optoelectronic properties of model systems of Ph_2_TDPP, EXG–TDPP, and c-EXG–TDPP (Methods and SI section 13). The first modeled system consists of a nanoflake of graphene (single layer graphene, or SLG) on which the oligomer, either monomer or dimer, is chemically bonded, thereby obtaining an interface labeled SLG–monomer and SLG–dimer (Figure 5a). This model system most closely resembles the EXG–TDPP system; Ph_2_TDPP results can be found in the Supporting Information. To model the SLG, a large nanoflake has been considered, in which only armchair edges saturated with hydrogen atoms are present. Upon formation of the covalent bond between the SLG and monomer, in which a radical species is present, an energy gap with a value of 4.56 eV is observed for both the SLG–monomer and SLG–dimer systems, which is 1 eV smaller compared to the oligomers considered alone (Figures S18 and S19). The main absorption peak of the SLG–dimer is red-shifted from the SLG–monomer case by 11 nm, which is relatively small compared to the much larger shift upon addition of the second covalent bond to another SLG reported later. The absorption spectra of the two interfaces are quite similar (Figure 5c, red and green curves), suggesting that the length of the oligomer only plays a minor role in the determination of the absorption properties of these interfaces. This comparison of monomer to dimer spectra is important, considering that we do not have the ability to control the oligomer length in the c-EXG–TDPP system. The broad, weak absorption peak at 600 nm (2.067 eV) can be directly related to the pristine nanoSLG but red-shifted by 100 nm and is due to a local transition within the graphene flake, from the SOMO to the LUMO. The intense peak observed at 470 (480) nm is purely due to the presence of the monomer (dimer) at the interfaces, with transitions originating from HOMO–2 to LUMO+1 (HOMO–2 to LUMO+2, Figure S19).

The next model system, SLG–monomer–SLG, built to study the differences between the nano-SLG, model oligomer, and cross-linked polymer, is shown in Figure 5a. Utilizing the optimized SLG–monomer structure described in the previous paragraph, a second SLG has been covalently bonded to the other tail of the oligomer, obtaining the final system. In the case of SLG–monomer–SLG and SLG–dimer–SLG, the SLG–SLG distance is 2.37 and 4.52 nm, respectively. The presence of the second SLG has a strong effect on the electronic properties of the interfaces, reducing the energy gap from 5.5 eV for the SLG–oligomer to 0.59 eV for the SLG–monomer–SLG system (Figure 5f); this result confirms that these cross-linked polymers are narrow band gap semiconductors. The underestimate of the band gap of c-EXG–TDPP could be due to the inclusion of only one sp^3^ defect per SLG flake formed at each TDPP covalent bonding site rather than, more accurately, multiple defects.^16^ Increasing the number of defect states would reduce the overall π-conjugation of the system. This band gap shift, along with calculated molecular orbitals (Figure 5b for example, Tables S3 and S4) demonstrate that CT states with electrons delocalized across multiple EXGs are responsible for the very large red-shift observed in the absorption spectra. For example, DFT shows here that there are multiple transitions (around 660–680 nm) between HOMO and LUMO+3 or LUMO+6 states for the SLG–monomer–SLG system (Figure 5b) with electronic population transferred from a state involving both nano-SLG and some character in the covalent-bound phenyl group to a state largely confined to one nano-SLG flake, with some phenyl character. Indeed, the HOMO and LUMO states show the radical character of the C–C bond between the sp^3^ carbon atom of the nanoflake and the phenyl atom of the Ph_2_TDPP monomer; orbitals with monomer character are found deeper in energy, namely HOMO–3 and LUMO+4 (Table S4). Yet, the molecular orbitals of this system are localized over fragments of the whole system, suggesting that CT proceeds from one SLG to another via a mechanism in which the monomer acts as a virtual bridge rather than a molecular wire.^71^

The optical properties are also affected by the presence of the second SLG. A strong red-shift of the absorption spectrum of the c-EXG–TDPP structure is observed, with a maximum absorption at 659 nm (1.882 eV) and a shoulder at 595 nm (2.084 eV), irrespective of the bridge oligomer length (Figure 5d); these results are in excellent agreement with the experimental spectra, with peaks observed at 661 (1.876 eV) and 630 nm (1.968 eV). In this case, we compare the c-EXG–TDPP structure to trimerized Ph_2_TDPP, because it is understood that c-EXG–TDPP system contains oligomeric chains of Ph_2_TDPP. Comparing to the Ph_2_TDPP trimer spectra, we observe that the strong peak of the SLG–monomer–SLG spectrum at around 500 nm is due to the oligomeric unit, while the bands red of 600 nm may be attributed to states involving the SLGs. In addition, the huge red-shift of about 160 meV strongly suggests that the oligomerization of the Ph_2_TDPP is not the main cause of the red-shift but is likely due to a more subtle CT process. Moreover, there is very little effect of the length of the oligomer on the absorption spectra in the cross-linked system. Thus, it seems that, in agreement with the orbital picture, the CT occurs from one EXG layer to another, with the organic molecule acting as a bridge, in a “superexchange-like” mechanism.^72−75^

The HOMO and LUMO states have strong localization around the sp^3^ carbon atom of the EXG, which has radical character. The density of states (DOS) suggest that the states responsible for the transitions are at lower occupied energies, in between −7/-5.5 eV (Figure 5e). The low energy, weak absorption peaks at around 1000 nm observed in the DFT results for both c-EXG–TDPP structures are likely due to the SLG–SLG transition with CT character, in view of the low oscillator strength, while the broad peaks at around 800 nm have some hybrid monomeric/dimeric-EXG character. Thus, we provide further evidence that the CT process happens with the help of the oligomer (strong absorption peak), acting as a bridge unit between “donor” and “acceptor” EXGs in which the CT states can be realized via broad transitions at low energy.

Finally, we report on analysis of the work functions (WF) for the investigated systems, which reveals more information regarding CT between the dye oligomers and nano-SLG. The system considered was the optimized monomer unit of the Ph_2_TDPP molecule without side chains to speed up computation, bound to one nano-SLG. Periodic boundary conditions are applied to simulate a monolayer of graphene.

The Fermi level of graphene has been computed here to be 4.46 eV, in excellent agreement with experimental data.^76^ The WF of the interface has a value of 4.14 eV, resulting in a shift of −0.32 eV from the pristine graphene. The negative sign of the shift suggests a decrease in the value for the electron injection barrier, which can be related to a downshift in energy of the LUMO level of the interface. The presence of the chemical bond at the interface leads to the formation of a radical species (Figure 5g). The projected density of states (PDOS) analysis confirms the localization of the singly occupied molecular orbital (SOMO) over the nanographene and its pinning at the Fermi energy of the interface. In addition, both HOMO and LUMO are localized over the oligomer (Figure S21). As a result, the different localization of the frontier orbitals gives rise to the observed CT process, with the SOMO localized over graphene and the LUMO over the molecule, indicating an electron transfer toward the molecule and eventually to the next graphene layer.

## Conclusions

In this article, we report on charge transfer dynamics in a cross-linked graphene/diphenyl-dithiophenediketopyrrolopyrrole nanohybrid following visible photoexcitation. These dynamics were revealed by global analysis of ultrafast transient absorption and two-dimensional electronic spectroscopy. In particular, the new charge transfer state is manifested as a transient photoinduced absorption feature located at 1.78 eV that forms within 18 ps and decays within 30–60 ps. Detailed DFT calculations were performed on model systems including monomer, dimer, and trimer Ph_2_TDPP, monomer/dimer EXG–TDPP, and SLG–monomer/dimer–SLG systems. These conclusive studies allow for careful comparison of the effects of bridge conjugation length and covalent bonding to graphene. We confirm the existence of a charge transfer process that proceeds via a superexchange mechanism between graphene–TDPP covalent bond sites, which may involve the transfer of charge density between covalently connected graphene sheets across the organic semiconductor bridges. This charge transfer process explains both the previously reported strong luminescence quenching and the strong red-shift in c-EXG–TDPP relative to the model Ph_2_TDPP molecule. This work will stimulate further efforts toward a deeper understanding of light-induced charge rearrangement processes in complex photoactive graphene-based nanohybrid architectures. Our comprehensive studies have demonstrated the great potential of the polymeric c-EXG–TDPP nanohybrid, which easily forms conductive thin films, as a novel solar energy harvesting platform.

## Supporting Information Available

Linear absorption and PL fitting, transmission electron microscopy images, additional transient absorption data, typical broadband pump pulse spectra used in 2DES experiments, all 2D DAS for Ph_2_TDPP and c-EXG–TDPP, time-resolution of 2DES experiments, anisotropy decay of Ph_2_TDPP in ps-TA, global analysis of EXG–TDPP ps-TA, additional ps-TA data in the NIR, analysis of oscillatory signals in Ph_2_TDPP and c-EXG–TDPP from 2DES experiments, global analysis details, photocurrent measurements on films, and additional calculations using density functional theory (PDF)
