# Supplementary material for: Deciphering Photoinduced Charge Transfer Dynamics in a Cross-Linked Graphene–Dye Nanohybrid
Source: J Phys Chem C Nanomater Interfaces. 2022 Feb 16;126(7):3569–81. doi: 10.1021/acs.jpcc.1c10570 (PMC8883522; doi:10.1021/acs.jpcc.1c10570)
Supplement: Supplementary file 1 — jp1c10570_si_001.pdf [file jp1c10570_si_001.pdf]

Supplementary information for "Deciphering Photo-induced Charge Transfer Dynamics in a Cross-linked Graphene-dye Nano-hybrid"

Aaron M. Ross,† Silvio Osella,‡ Veronica R. Policht,† Meng Zheng,¶ Michele Maggini,¶ Fabio Marangi,†,§ Giulio Cerullo† Teresa Gatti,‖ and Francesco Scotognella†,§

†Department of Physics, Politecnico di Milano, Piazza Leonardo da Vinci 32, 20133, Milano, Italy
‡Chemical and Biological Systems Simulation Lab, Centre of New Technologies, University of Warsaw, Banacha 2C, 02-097 Warsaw, Poland
¶Chemical Sciences Department, Università degli Studi di Padova, Via Marzolo 1, 35131, Padova, Italy
§Center for Nano Science and Technology, Istituto Italiano di Tecnologia, Via Pascolo, 70/3 Milano 20133, Italy
‖Center for Materials Research, Justus Liebig University, Heinrich-Buff-Ring 17, 35392, Giessen, Germany

Section 1: Linear absorption and emission fitting

		**Ph2TDPP**					
		**Absorption**			**Emission**		
**Manual**	Peak (eV)	2.412	2.210	2.077	2.000	1.829	1.664
**Fit**	Peak (eV)	2.318	2.184	2.052	1.999	1.840	1.680
	Width (eV)	0.109	0.074	0.052	0.052	0.062	0.082
		**EXG-TDPP**					
		**Absorption**			**Emission**		
**Manual**	Peak (eV)	2.360	2.159	2.018	1.949	1.841	1.665
**Fit**	Peak (eV)	2.391	2.136	1.989	1.947	1.800	1.665
	Width (eV)	0.166	0.125	0.066	0.057	0.064	0.103
		**c-EXG-TDPP**					
		**Absorption**			**Emission**		
**Manual**	Peak (eV)	2.17	2.00	1.84	N/A		
**Fit**	Peak (eV)	2.22	1.98	1.82			
	Width (eV)	0.22	0.13	0.09			

Table S.1: Peak energies and widths of linear absorption and photoluminescence (emission) determined both manually and using least squares non-linear fitting for Ph2TDPP, EXG-TDPP, and c-EXG-TDPP. In the manual case, the peaks were determined by averaging the local maxima of the signal, the zero crossings of the first derivative, and the minima of the second derivative for each spectrum. In case of least squares non-linear fitting, for absorption, 5-7 Gaussian functions were used, while for emission, three Gaussians were fit to the spectra. Only the three resulting peak

energies in the vicinity of the relevant transient absorption energy range, and the suspected 0-0 transition energies for each molecule are shown here. The widths are standard deviations in the Gaussian function, so that FWHM = $2\sqrt{2ln2}\sigma$.

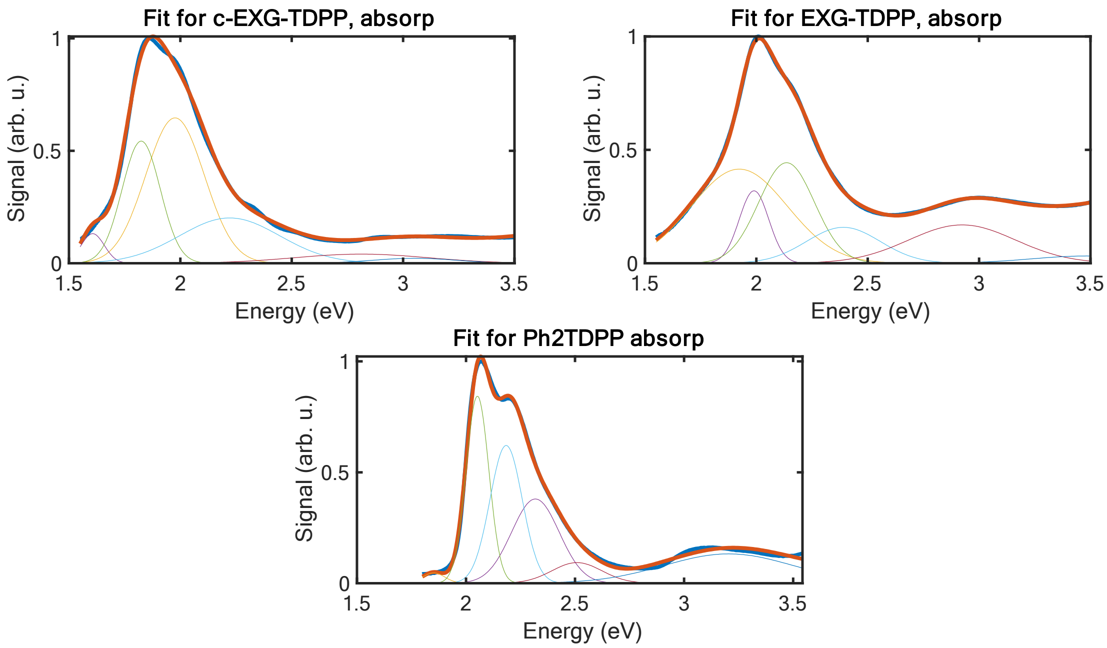

Figure S.1: Linear absorption spectra fits resulting in parameters given in Table S.1. Blue curve is data, red curve is the fit, lighter curves are the individual Gaussians.

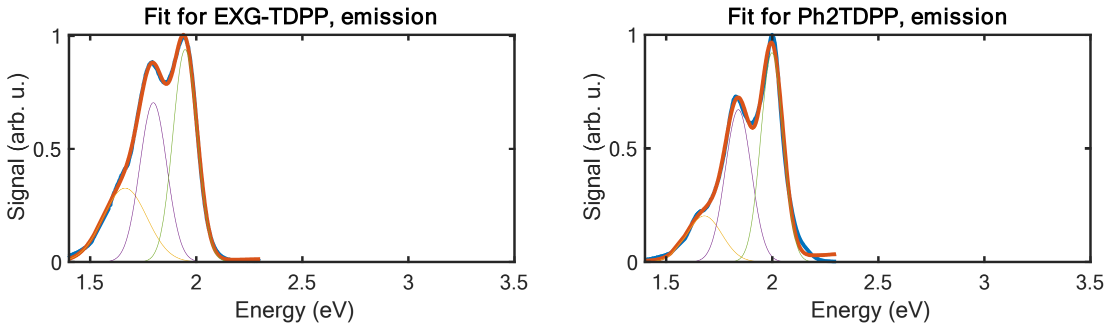

Figure S.2: Emission spectra fits for Ph2TDPP and EXG-TDPP resulting in parameters given in Table S.1. Blue curve is data, red curve is the fit, lighter curves are the individual Gaussians.

Section 2: Transmission electron microscopy

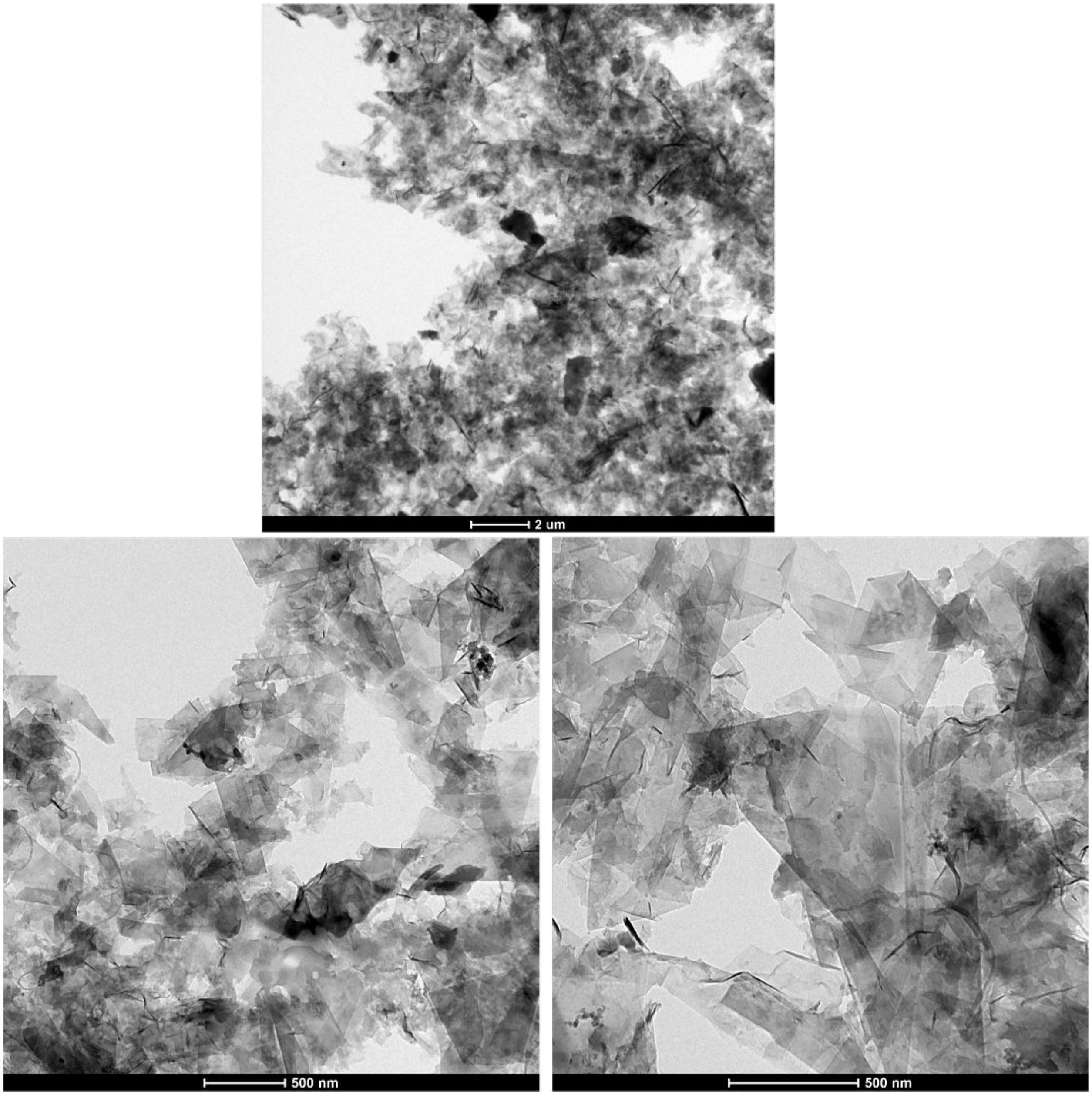

Figure S.3: Transmission electron microscopy images of c-EXG-TDPP. Low (top) and high (bottom) magnification images are shown. The presence of crystalline graphene nanosheets and amorphous areas due to the presence of oligomeric TDPP units is well distinguished in the images.

Section 3: Additional transient absorption data

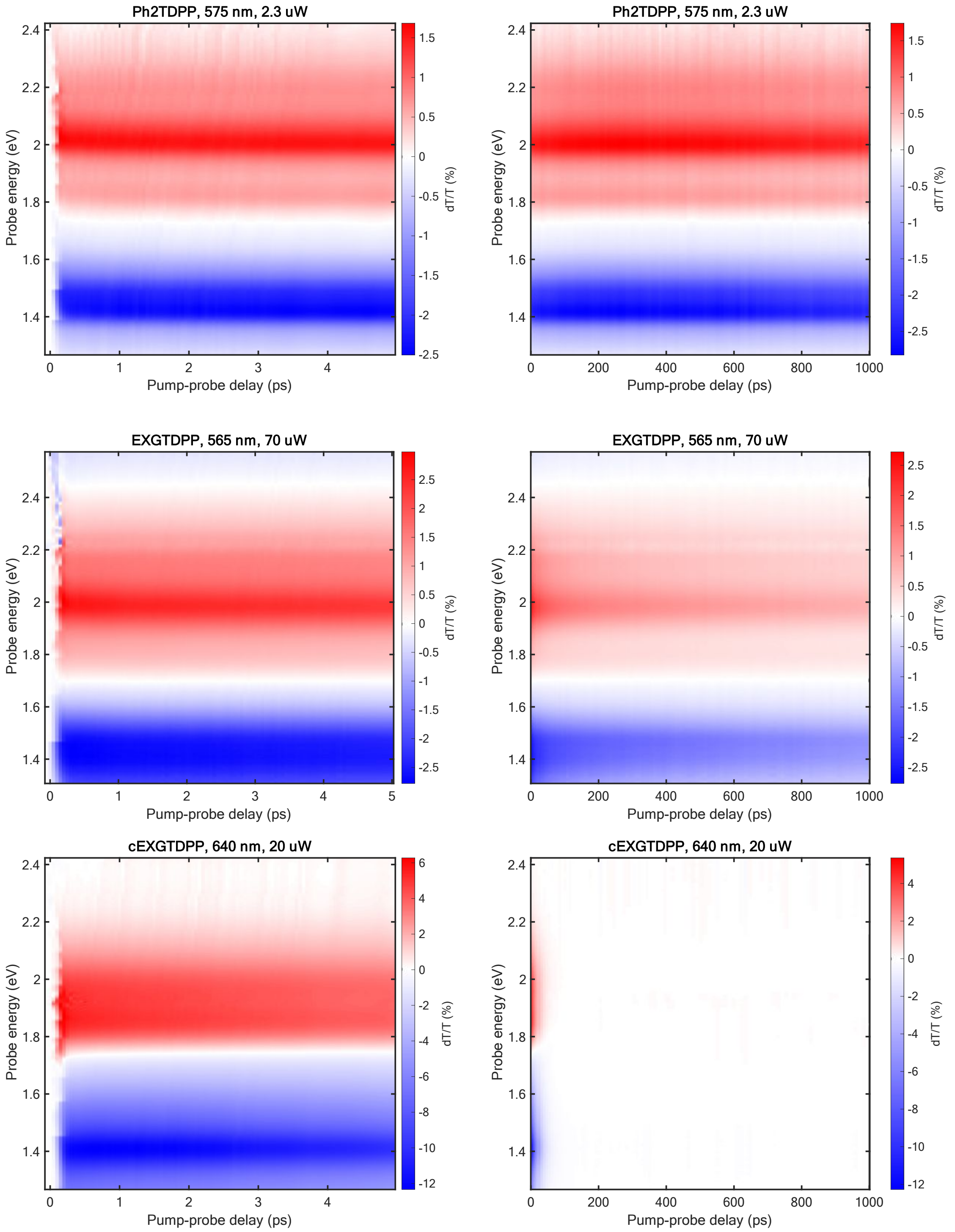

Figure S.4: Transient absorption maps for Ph2TDPP, EXG-TDPP, and c-EXG-TDPP. Left column: -1 ps to 5 ps; Right column: -1 to 1000 ps. The sample type, pump wavelength, and average pump power are indicated in the titles of each plot.

Section 4: Typical broadband pump pulse spectrum used in 2D spectroscopy experiments

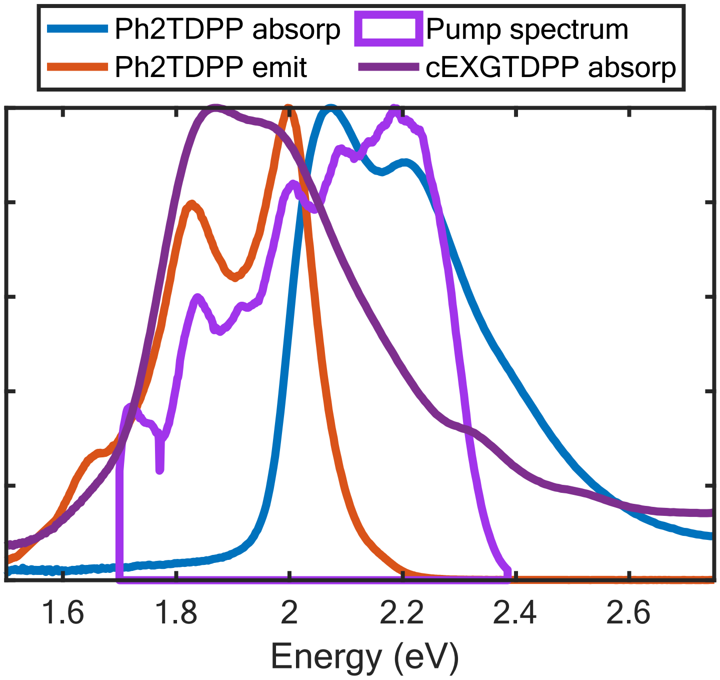

Figure S.5: Typical pump spectrum used in 2D spectroscopy experiments, overlaid on linear absorption and emission spectra of Ph2TDPP and cEXGTDPP. The pump spectrum (purple with shaded area underneath of curve) is derived here taking the Fourier transform of the pump-pump interferogram measured on a photodiode as a function of t_1, which is used for calibrating the pump energy axis [1]. This spectrum changes on a day-to-day basis depending on how the NOPA is aligned, but does not vary much from what is shown in this figure. Linear absorption (blue) and emission (red) spectra for Ph2TDPP and the absorption (dark purple) spectra for cEXGTDPP are also shown. The absorption spectra are divided by the energy, and the emission spectra are divided by the energy cubed [2].

Section 5: All 2D DAS for Ph2TDPP and c-EXG-TDPP

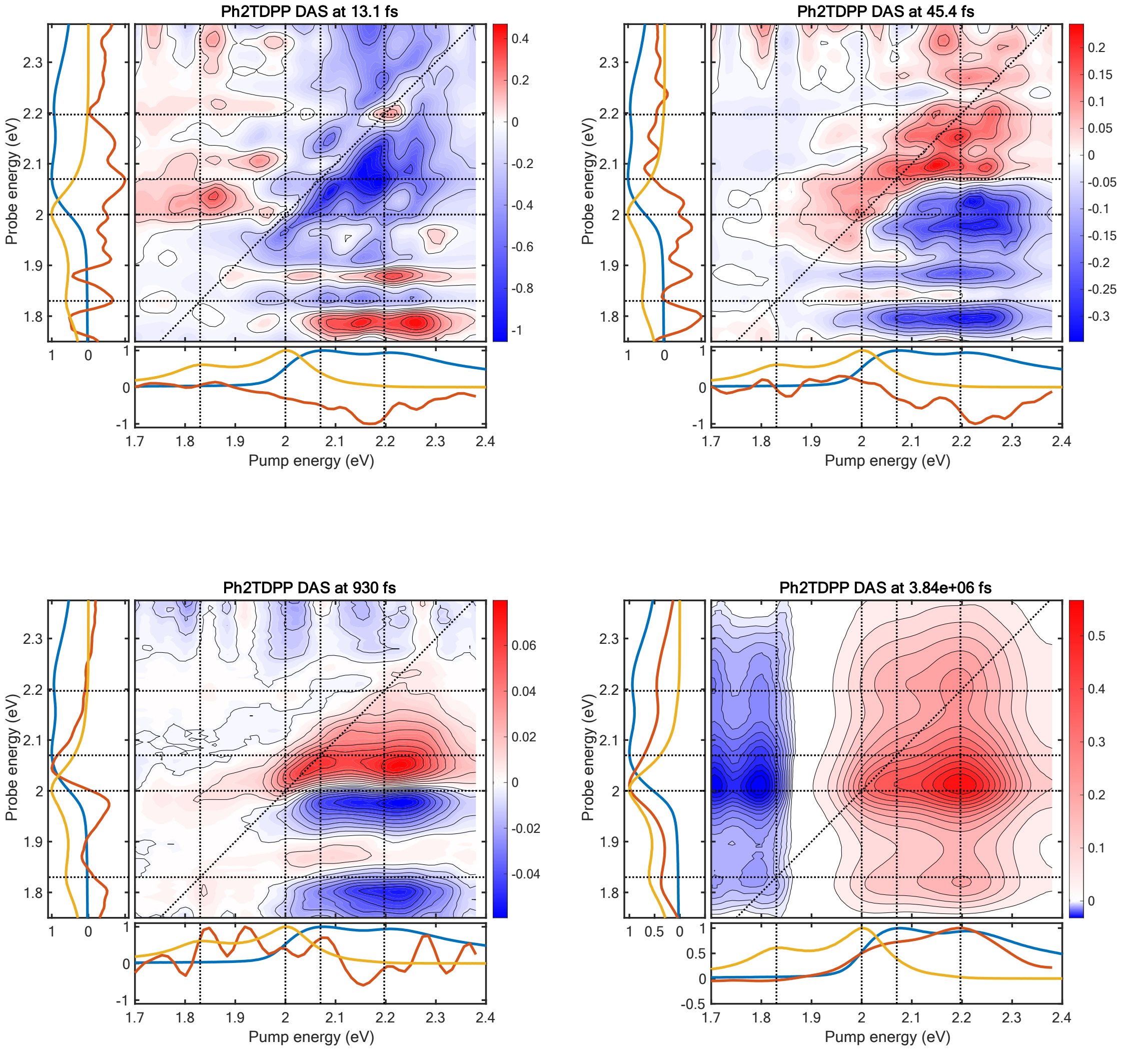

Figure S.6: All 2D DAS for Ph2TDPP experiment, with T2 scanning from -100 to 2000 ps in 10 fs steps. Lifetimes are indicated in title above each map.

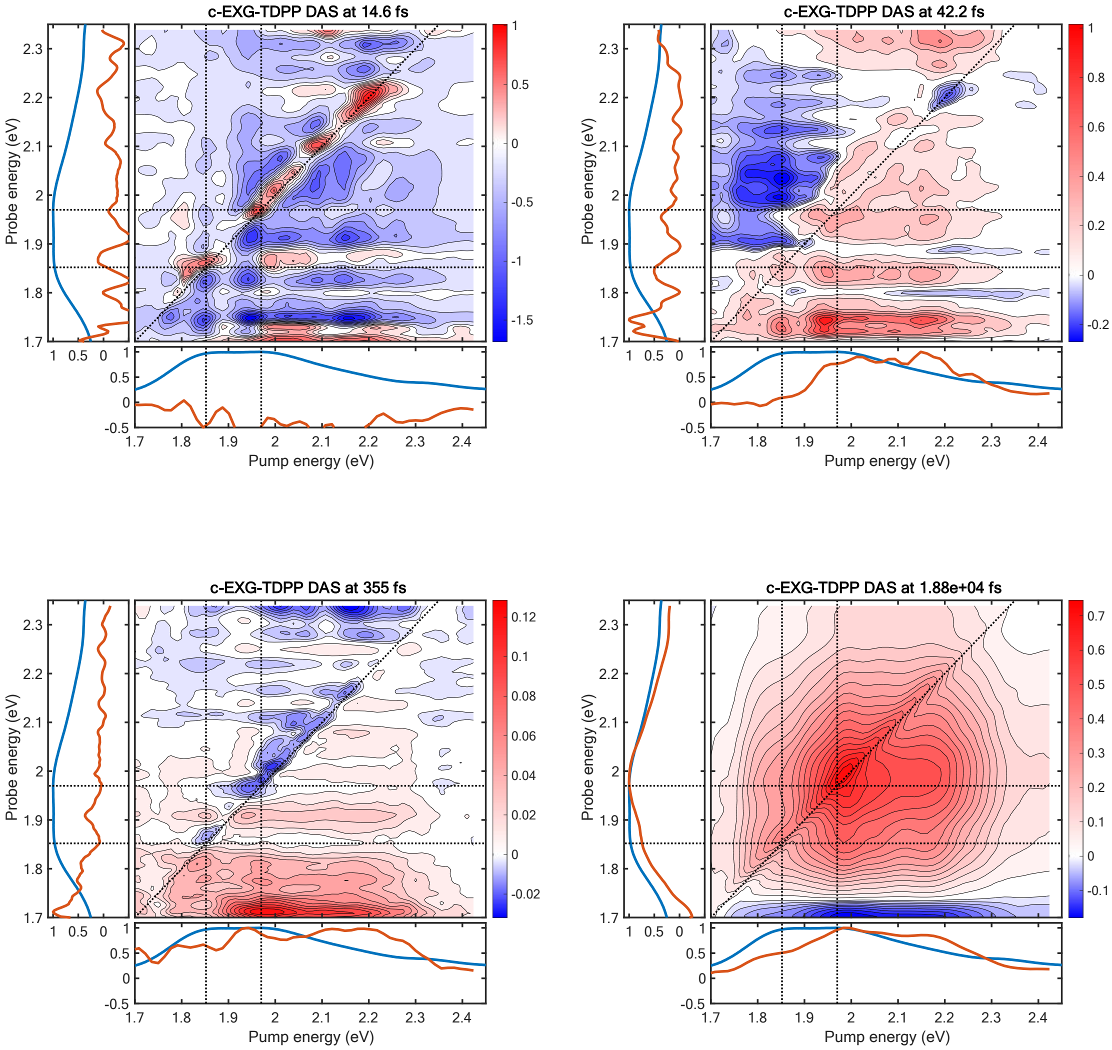

Figure S.7: All 2D DAS for c-EXG-TDPP experiment, with T2 scanning from -100 to 2000 ps in 10 fs steps. Lifetimes are indicated in title above each map.

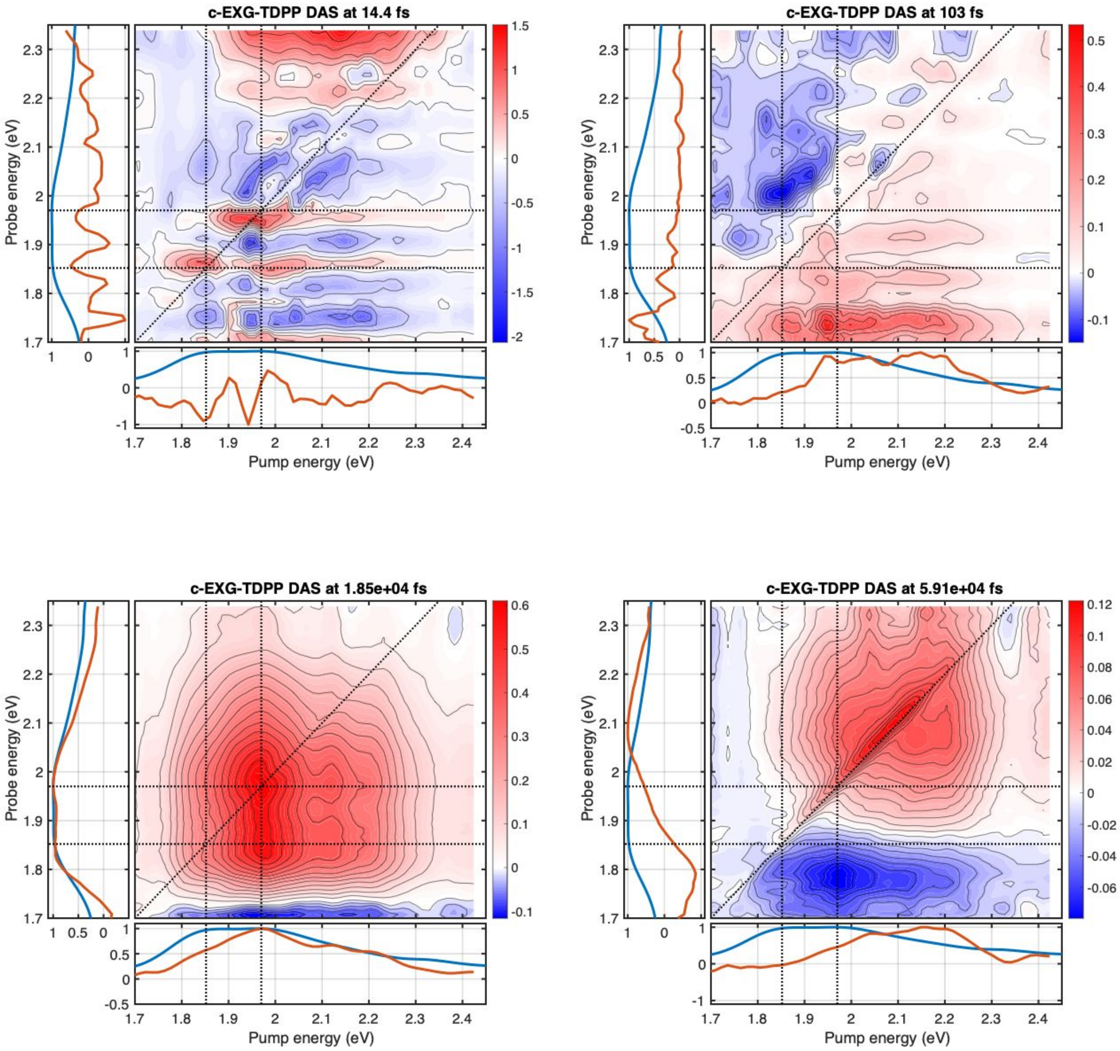

Figure S.8: All 2D DAS for c-EXG-TDPP experiment, with T2 scanning from -100 to 60 ps. Lifetimes are indicated in title above each map.

Section 6: Time-resolution of 2D experiments

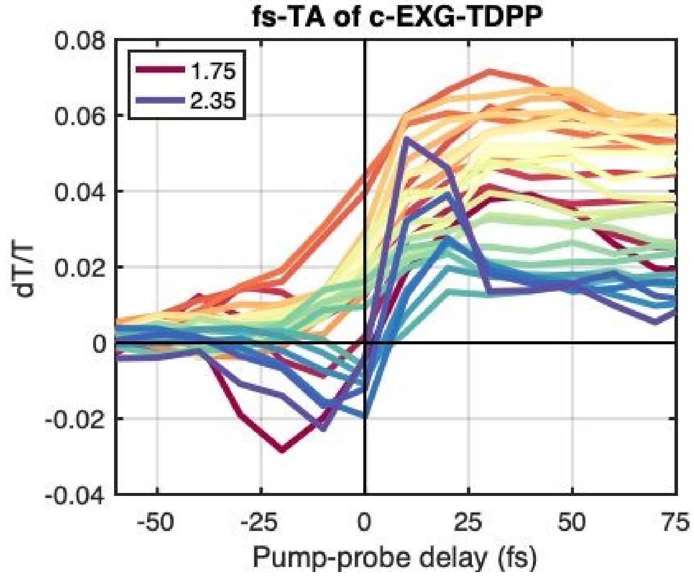

Figure S.9: Transient absorption taken with same pump and pulse spectra utilized in c-EXG-TDPP 2D experiments. A large number of kinetic traces are shown for probe energies ranging from 1.75 to 2.35 eV (with only the first and last colors shown in legend, interpolating color between the two). The rising edge of each trace is nearly simultaneous within a 25 fs range around zero.

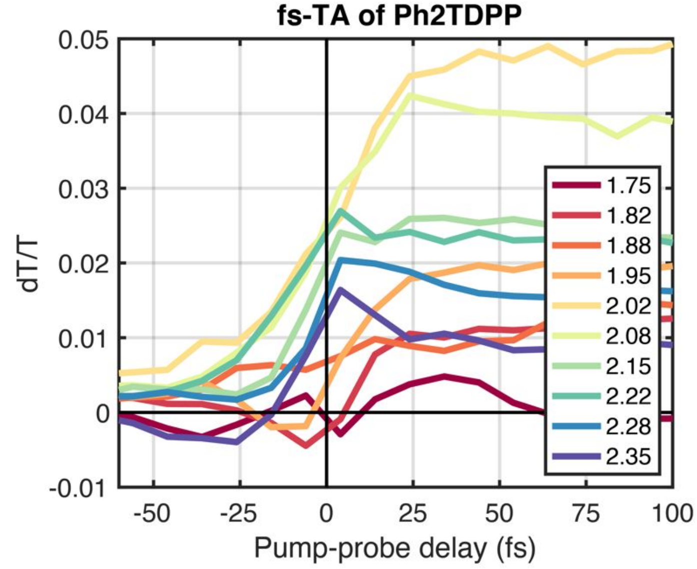

Figure S.10: Transient absorption taken with same pump and pulse spectra utilized in Ph2TDPP 2D experiments. A large number of kinetic traces are shown for probe energies ranging from 1.75 to 2.35 eV (indicated in the legend).

Section 7: Anisotropy decay of Ph2TDPP in ps-TA

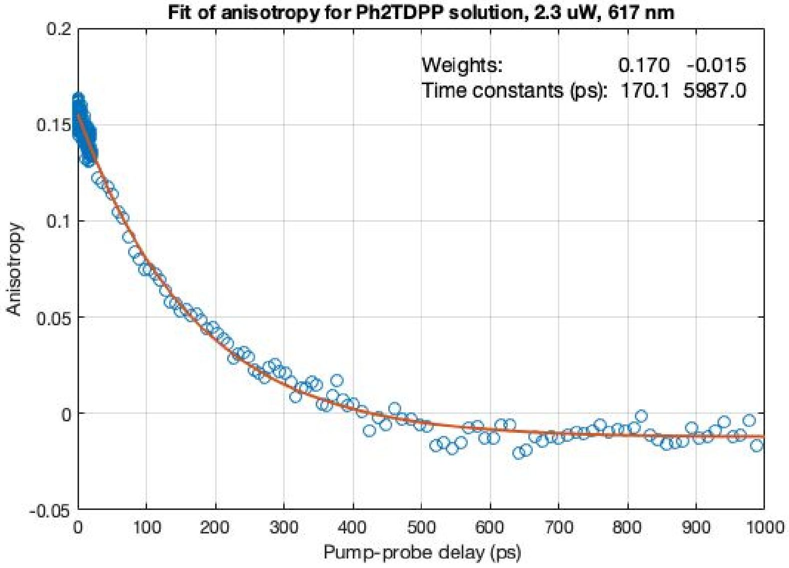

Figure S.11: Anisotropy decay of Ph2TDPP solution, probing at 617 nm, pumping at 575 nm

A time-dependent anisotropy measurement was performed in Ph2TDPP solution by utilizing both cross- and co-polarized pump and probe configurations. When the pump is cross-polarized relative to the white light probe, the broadband TA signal is reduced overall during the 137 ps lifetime determined by global analysis, while the signal is enhanced in the co-polarized configuration (not shown). This pump polarization dependence is indicative of time-dependent anisotropy in the solution-dispersed Ph_2TDPP system, which reflects dynamics associated with alignment of the transition dipole moments of the molecule, or molecular orientation randomization due to rotational diffusion [3, 4, 5]. The anisotropy term r(t), given by

$$r(t) = \frac{S_{\parallel}(t) - S_{\perp}(t)}{S_{\parallel}(t) + 2S_{\perp}(t)}$$

where $S_{\parallel/\perp}(t)$ is the time-dependent TA signal under co/cross-polarization excitation conditions. The anisotropy decay for Ph_2TDPP was fit with the sum of two exponentials, arriving at lifetimes of 170.2 ± 15.2 ps, and > 3 ns, with weights of 0.17 and -0.015, respectively. The fast anisotropy decay is nearly agreement with the global analysis DAS that flips sign with the pump polarization. However, for cEXGTDPP, no time-dependent anisotropy or pump-polarization-dependent kinetics are observed (not shown); however, the anisotropy near zero pump-probe delay is the same as in Ph_2TDPP (~0.15). The anisotropy decay in cEXGTDPP may not be measurable here due to the rapid decay of the signal (mostly decayed with 20 ps) compared to the 170 ps anisotropy decay in Ph2TDPP.

Section 8: Global analysis of EXG-TDPP ps-TA

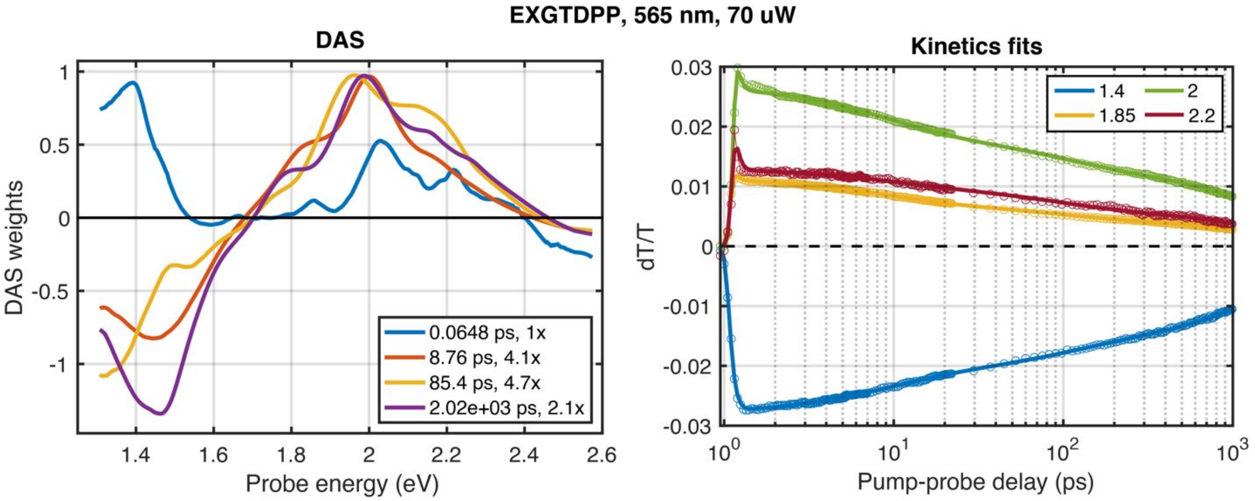

Figure S.12: Global fitting results for picosecond transient absorption of EXG-TDPP in toluene solution. The sample was pumped at 565 nm with 70 µW average power. *Left:* The four decay-associated spectra are shown, with corresponding lifetimes and factors by which the DAS are multiplied in the plot shown in the legend. *Right*: Selected kinetics (empty circles) and fits (solid lines) at selected probe energies (see legend).

Section 9: YAG NIR white light probe, 400 nm pump ps-TA

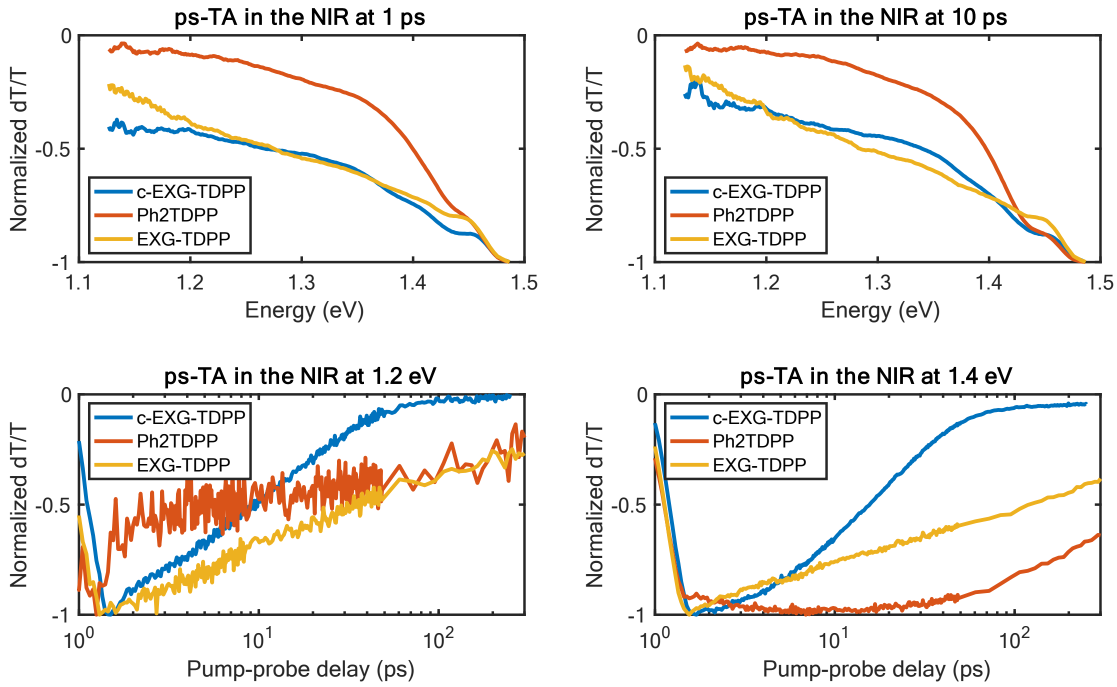

Figure S.13: Comparison of pump-probe results in the NIR (1.15-1.5 eV probe energy range) when pumping at 400 nm for Ph2TDPP, EXG-TDPP, and c-EXG-TDPP. Top left: dT/T spectra at 1 ps pump-probe delay time. Top right: dT/T spectra at 10 ps pump-probe delay time. All spectra are normalized by the absolute value of the maximum of the spectrum, for easy comparison since the EXG-TDPP and c-EXG-TDPP signals are much smaller than Ph2TDPP. Bottom left (right): kinetics at 1.2 eV (1.4 eV) probe energy, again normalized for easy comparison.

Section 10: Analysis of oscillatory signals in Ph2TDPP and c-EXG-TDPP

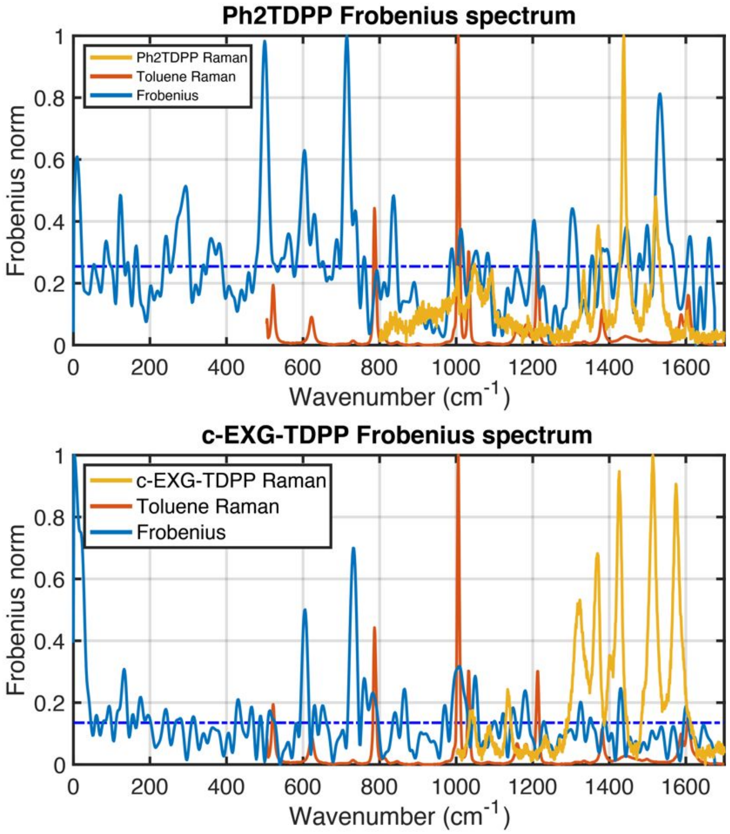

Figure 14: Frobenius spectra for Ph2TDPP and cEXGTDPP, overlaid on sample Raman scattering [6] and toluene Raman scattering [7].

After global fitting of the 2D data, the residuals of the fits may be examined for coherent oscillations. One of the first steps in the coherent analysis process is to take the Frobenius norm [8], which provides an easy-to-interpret presentation of the power spectrum of all oscillatory modes present in the 2D data. While a large number of coherent oscillations were observed in Ph2TDPP due to large signal strength, oscillatory modes were not easily observed in c-EXG-TDPP likely due to significant pump scattering and NOPA spectrum instabilities. The coherence analysis is also complicated by the presence of toluene modes, which have been shown previously to contribute to oscillatory signals, which may either be superimposed on the Ph2TDPP/cEXGTDPP signal, or interact via general solvent-solute interactions [9]. The coherent oscillations observed here in Ph2TDPP/cEXGTDPP systems will be studied in greater detail with different solvents in the future.

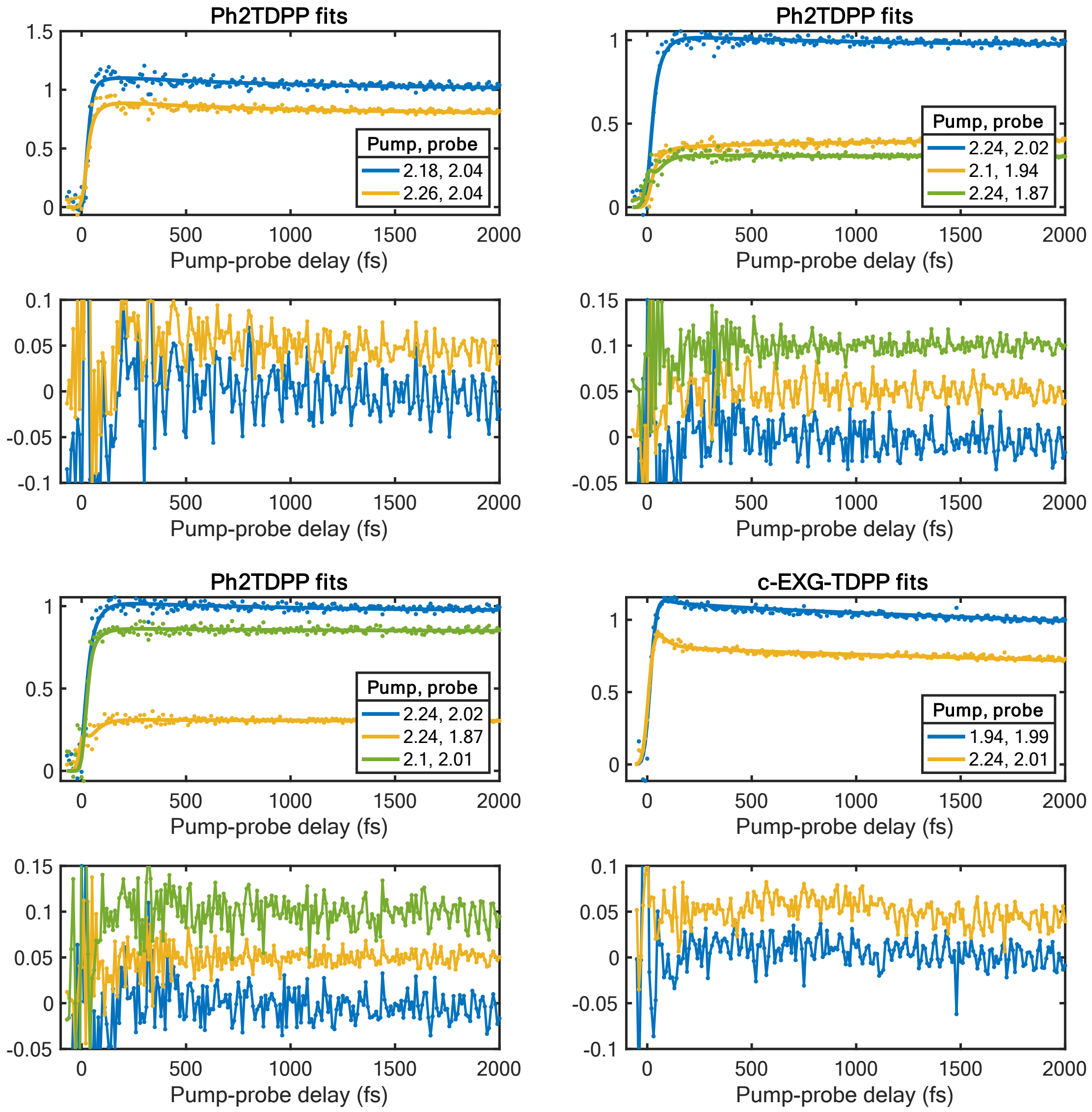

Figure 15: Selected fits and residuals of Ph2TDPP (plots 1-3)/ cEXGTDPP (plot 4) fs-2D data. Pump/probe energies were chosen to show significant oscillatory behavior at the following modes, respectively in the first, second, third, and fourth plot: 292, 716, 1536, 605.3 cm^-1.

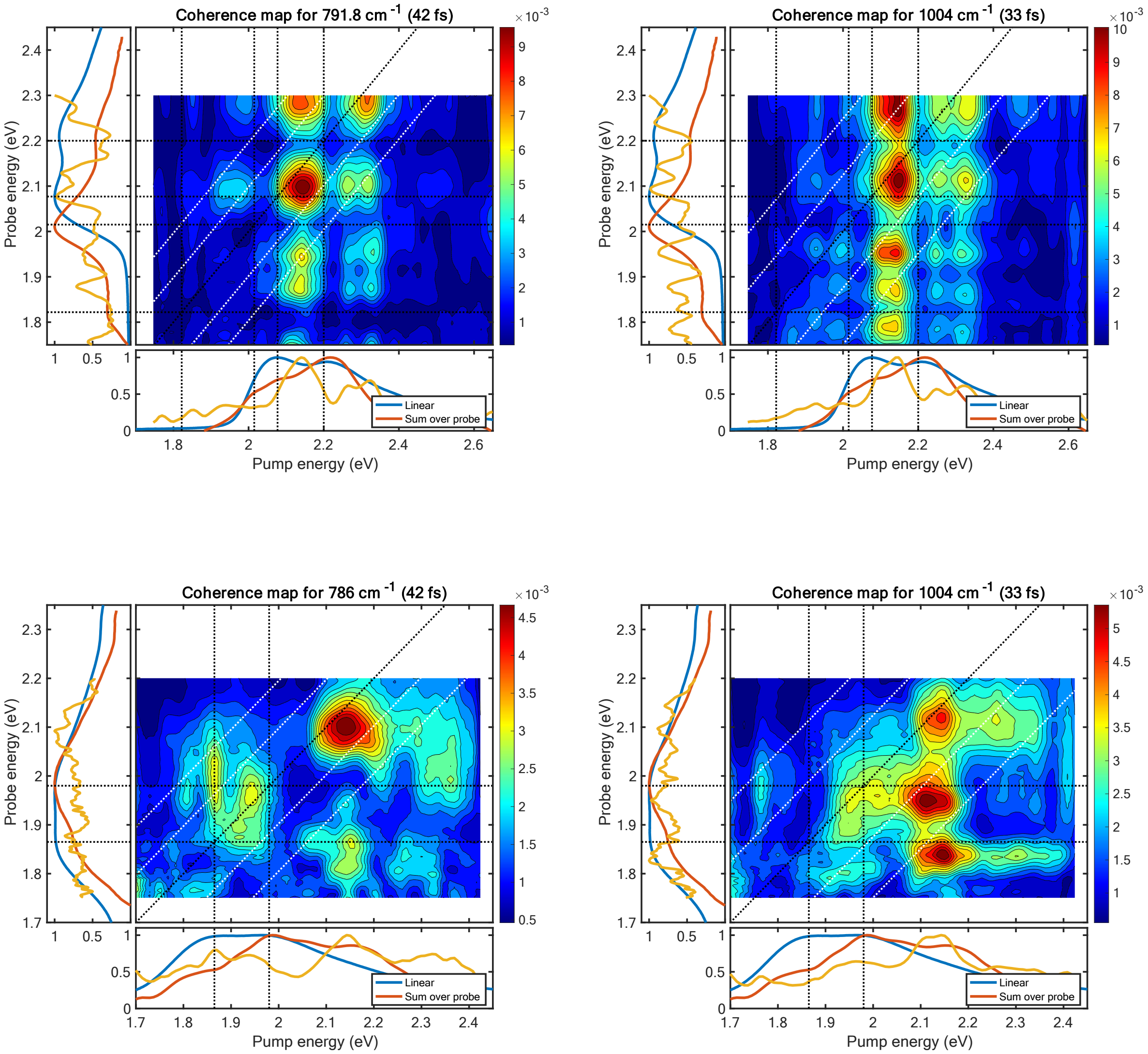

Figure 16: Coherence maps for Ph2TDPP (top figures) and cEXGTDPP (bottom figures) around 790 cm^-1 and 1004 cm^-1. Black vertical and horizontal lines correspond to resonance energies determined by steady state emission and absorption, black diagonal dashed line corresponds to $E_{pump} = E_{probe}$, and the white dashed diagonal lines correspond to $E_{probe} = E_{pump} \pm n\hbar\omega$ where ω is the coherence frequency (either 790 or 1004 cm^-1).

Here the coherence maps for Ph2TDPP and cEXGTDPP are shown for specific strong signals in the Frobenius spectra that overlap with toluene Raman modes. These maps are determined first by subtracting the kinetics determined by global fitting at all pump/probe energies. Then, at each pump energy, the signal is fast Fourier transformed, thus converting the 3D $\omega_1 - t_2 - \omega_3$ data set into a $\omega_1 - \omega_2 - \omega_3$ data set, where ω_2 is now the coherence frequency. The absolute value of the FFT is shown.

We consistently observed strong resonances when pumping between 2.1 and 2.2 eV in both samples. It is noted in the 1004 cm^-1 mode in cEXGTDPP that strong resonant features are observed where the white diagonal lines overlap with the BG2 and BG1 linear absorption resonances at 1.98 and 1.85 eV. Notable resonance structure [8, 10] is also observed at 786 cm^-1 for Ph2TDPP. At this time, we tentatively note that this pump energy coincidentally aligns with the center of the GSB in cEXGTDPP that decays with a lifetime of around 50 ps.

The interaction between Ph2TDPP/c-EXG-TDPP and the solvent, in this case toluene, likely plays a strong role in determining the efficiency of charge transfer [14, 15, 9]; however, the role of the vibrational modes of the solvent as related to charge transfer is not well understood. It is noted here that the coherence analysis shows that toluene vibrations may be enhanced by resonance conditions that are satisfied in both Ph2TDPP and c-EXG-TDPP. In particular, strong toluene oscillations were observed when pumping around 2.15 eV in both systems, which is coincidentally aligned with the GSB of the subset of states in the >50 ps 2D-DAS of c-EXG-TDPP. At this time, we thus conclude that toluene could play a role in the charge transfer observed here, or at least serves as a spectator of charge transfer.

Section 11: Global analysis

The main data analysis technique used throughout this paper is known as global analysis [11]. In this analysis, it is assumed that the kinetics measured via pump-probe and 2DES may be described by the sum of a finite number of exponential decays that have been convolved with the instrument response function. Each of the exponential decays are associated with a physical process, more specifically the decay/filling of populations in the system. In the case of pump-probe, a fixed number of decays are assumed, and only the coefficients (weights) in front of each exponential term are allowed to vary as a function of probe wavelength.

More explicitly, the pump-probe signal *S* is fit with the following functional form,

$$S(\lambda,\Delta t) = \mathrm{IRF} * \sum_{j=1}^{N} A_j(\lambda) e^{\frac{-\Delta t}{\tau}}$$

where IRF is the instrument response function, which is taken to be a Gaussian function with the FWHM of the pump pulse, is convolved (*) with the sum of weighted exponential decays with lifetimes τ. Δt is the pump-probe delay, and λ is the probe wavelength. The probe wavelength-dependent coefficients $A_j(\lambda)$ are referred to as decay-associated spectra (DAS). These DAS, along with the lifetimes, decompose the pump-probe signal into a finite number of spectra which with further modeling provide a connection between these spectra and physically meaningful processes.

It is important to note that, without further modeling and interpretation, that the resulting DAS must be taken to add together simultaneously. For instance, an increase of an excited state population, as observed via the development of a stronger negative dT/T signal (ESA) at later pump-probe delay times, could be represented by the sum of a fast positively-weighted exponential (decay of a bleaching state, in-filling of excited state) and a slower negatively-weighted exponential (decay of the excited state).

Extension of the global analysis technique to 2DES is conceptually simple, but technically demanding [12]. The 2DES data is "unfolded" into a 2D array with the number of rows equal to product of the number of probe and pump energies, and the number of columns equal to the number of t2 points (waiting time between second pump pulse and probe pulse). Then, the DAS become functions of both the pump and probe energy, allowing us to break down the 2DES signal into physically-interpretable DAS in the same manner as in the pump-probe experiments. However, a few points must be noted. First, only exponentials with real arguments (decays) are allowed; other workers have extended this technique to include oscillatory signals [12]. In our coherence analysis, we simply examine the residuals of the signal after subtracting the kinetics fit via global analysis. Second, the data sets are intractably large to fit with plug-and-play non-linear least squares packages from *Matlab*. Thus, we developed a home-built 2D global fitting package in *Matlab* that succeeded in fitting these large data sets, taking advantage of two features: 1. Reduction of the data set size: while the number of t2 points and pump points are kept the same as in the experimental data set, we sample 40-60% of the original

probe points. 2. Direct analytic calculation of the Jacobian of the minimization function used in the non-linear least squares procedure. This second feature involves the utilization of sparse matrices, which leads to huge memory savings in the calculation, making this fitting problem tractable on a personal computer. Further details will be provided in a future paper, with source code available upon request.

Section 12: Film photocurrent measurements

It has been reported previously [6] that spin-coated films of c-EXG-TDPP on ITO substrates exhibit conductivity with a nearly-Ohmic current-voltage curve (~50 kOhm), compared to negligible conductivity for thin films of Ph2TDPP. This conductivity certainly arises due to the presence of graphene in thin films, known for its exemplary transport properties [13]. However, it is not yet understood if charge separation occurs in thin films of c-EXG-TDPP under optical excitation; charge separation could be utilized for potential solar cell/photodetector applications.

Here we report on the results of steady-state and transient photocurrent experiments on thin films of c-EXG-TDPP and Ph2TDPP drop-casted onto glass substrates with ITO interdigits. These ITO interdigits are used to provide an electric field across the drop-cast films to extract photocurrent.

In order to measure photocurrent generated in c-EXG-TDPP films drop-cast on interdigitated ITO substrates, the NOPA utilized to produce pump pulses for the TA experiments was tuned to 645 nm, near the main absorption resonance. In the steady-state experiment, every other pump pulse was blocked by a mechanical chopper at 500 Hz; the chopper reference was sent to a lock-in amplifier. The pump pulses were focused onto the sample with a 240 μm spot size diameter, with the focus centered in the 50 μm wide channels between ITO interdigits. The photocurrent was collected across two larger ITO pads, converted to a voltage via a transimpedance amplifier with gain set to 10^7 V/A, and sent to the lock-in amplifier. A sample bias voltage of 10 V was placed across the ITO interdigits, resulting in an electric field of ~2000 V/cm. Background photocurrent was measured by translating the sample position to allow the pump to focus on a section of c-EXG-TDPP film not cast between ITO interdigits, thereby not collecting photocurrent; the background result was zero, within the signal to noise ratio of the experiment. Additionally, no photocurrent was measured under excitation of the ITO interdigits without c-EXG-TDPP film.

A detectable responsivity of 18 mA/W was measured under 100 fs pulse excitation at a 500 Hz repetition rate with a central wavelength of 645 nm; under assumptions of complete absorption of incoming radiation and total collection of photocurrent that reaches the ITO contacts, we arrive at an average of 0.03 electrons/incident photon for the c-EXG-TDPP thin film. In fact, we likely underestimate the number of electrons generated per photon, since the channel width is 50 μm and the beam spot diameter is around 240 μm, leading to illuminated inactive regions of the absorbing medium lying on top of the ITO contacts.

The photocurrent measurement was checked by comparing the photocurrent reading to: 1. Optical excitation of ITO contacts only, 2. Optical excitation of c-EXG-TDPP deposited next to the ITO contacts but outside of non-zero electric field regions, 3. Optical excitation of Ph2TDPP deposited between ITO contacts on the same substrate, 4. Blocked optical excitation to measure leak-through current. None of these experiments produced a detectable photocurrent on the lock-in amplifier. It is noted that photocurrent was only

observed under relatively high field strengths of around 2000 V/cm, that the photocurrent was heterogeneous over the drop-casted sample as indicated by variations in signal strength as the photo-excited sample position was scanned, and that photocurrent degraded due to high excitation fluences over the course of less than an hour. Sample position variations lead to responsivities ranging from 0 to 18 mA/W.

Thus, we report that simple drop-casting deposition of c-EXG-TDPP yielding a thin film between ITO contacts results in a measurable photocurrent under ultrafast optical excitation, with approximate quantum efficiencies of ~3% (0.03 e/photon). This photocurrent measurement implies that ultra-fast charge separation is occurring in the solid-state, and that this charge separation can be harvested along the graphene scaffolding in a thin film. The authors point out that the solid-state system studied via photocurrent measurements presented here differ significantly from the toluene-dissolved c-EXG-TDPP system discussed primarily in the main body of the paper. It is also well-known that solvents play an outsized role in charge transfer processes in solution. The purpose of this section is to demonstrate charge transfer in the solid state c-EXG-TDPP system, thereby providing further feasbility for the hypothesis of charge transfer in the toluene-dissolved c-EXG-TDPP system in the main body of the paper. Furthermore, we do not present in any detail the results of transient absorption studies on film samples of Ph2TDPP and c-EXG-TDPP in this paper.

Section 13: Additional calculations from density functional theory

A preliminary benchmark of different long-range corrected functional has been performed in order to assess the correctness of the opto-electronic description of the investigated systems. The Ph2TDPP monomer has been considered for benchmark and its geometry optimized with seven different funcitonals: CAM-B3LYP, CAM-B3LYP-D, wB97xD, lc-wHPBE, M06-D, lc-wPBE-D and HSE. The total energy has been considered as key parameter. After geometry optimization, the lowest total energy was obtained with the wB97XD functional, which therefore was chosen for the study. All calculations were performed with this functional and the 6-31G(d,p) Pople basis set, within the Gaussian16 software.

Electronic properties of Ph2TDPP monomer-trimer:

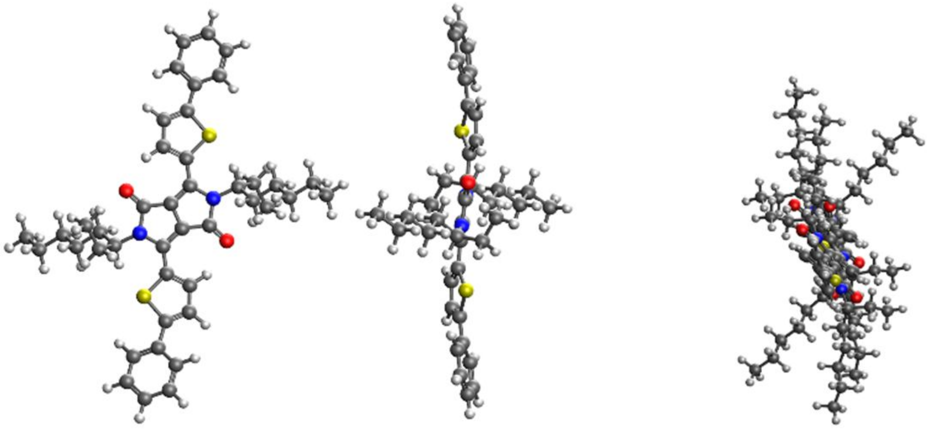

Figure 17: Geometries of the Ph2TDPP monomer used in DFT calculations. Left: Ph2TDPP monomer from front, right: Ph2TDPP monomer from side.

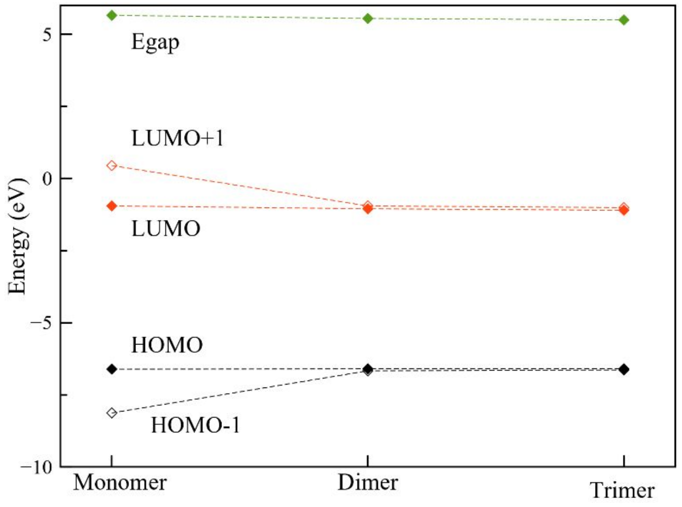

Figure 18: Energy gap diagram for Ph2TDPP monomer, dimer and trimer.

The electronic properties of the oligomers of Ph2TDPP (up to the trimer) are reported in Figure 18. It is clear from this analysis that already at the trimer level the HOMO and LUMO energies as well as the energy gap are converging towards the polymer values. The HOMO and LUMO are lying at a similar energy of -6.60 and -1.10 eV for both dimer and trimers, leading to an energy gap of 5.50 eV, to be compared with the bandgap for the polymer (obtained from the monomer geometry) of 5 eV. Interestingly, it seems that the increase of conjugation does not strongly affects the energy gap, which might be due to the twist in the molecular structure of the oligomers.

Absorption spectra of Ph2TDPP oligomers

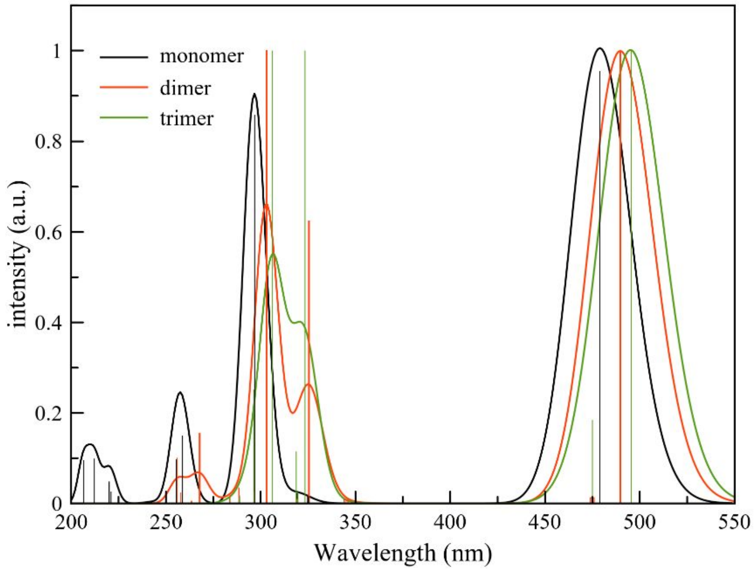

Figure S.19: Absorption spectra for Ph2TDPP monomer (black), dimer (red) and trimer (green).

**Monomer**	**Wavelength (o.s.)**	**transition**	**FMO (frontier molecular orbital)**	
S1	479 (0.96)	H → L	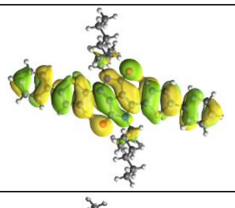	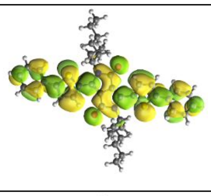
S5	297 (0.86)	H-2 → L	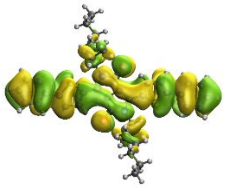	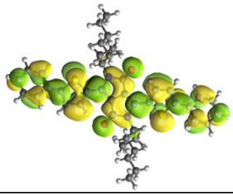

S8	259 (0.15)	H → L+2	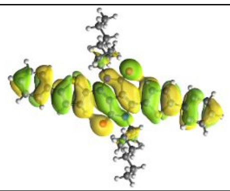	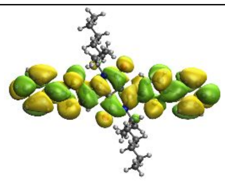
**Dimer**	**Wavelength (o.s.)**	**transition**		
S1	490 (2.41)	H → L	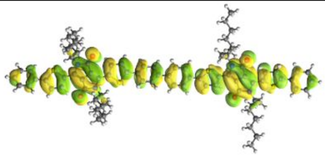	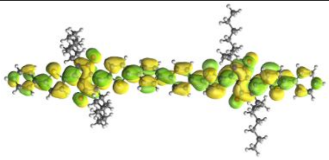
S5	325 (0.62)	H → L+2	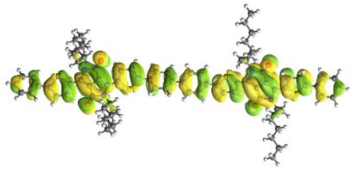	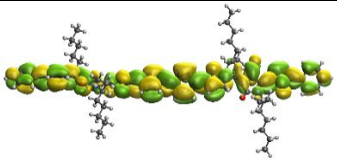
		H-2 → L	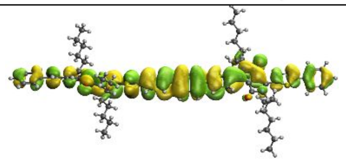	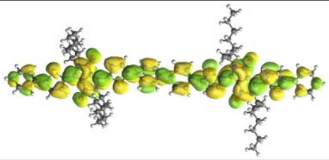
S9	303 (1.59)	H-4 → L	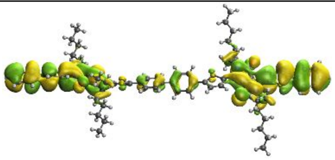	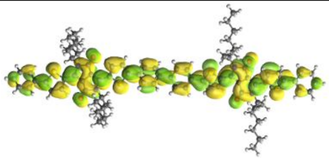
**Trimer**	**Wavelength (o.s.)**	**transition**		
S1	496 (3.78)	H → L	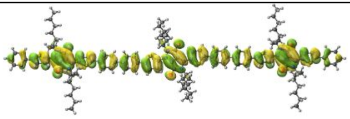	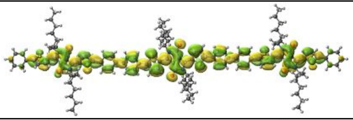
S8	323 (1.29)	H-4 → L	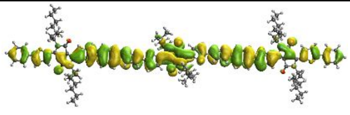	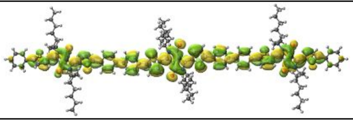
S13	306 (1.97)	H-1 → L+5	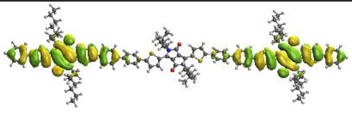	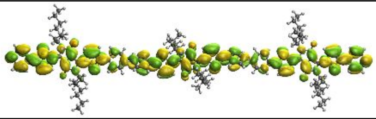

Table S.2: Long wavelength transitions for the Ph2TDPP monomer, dimer, and trimer systems calculated from DFT.

SLG-oligomers

The system consists of a nanoflake of graphene (nano-SLG) on which the oligomer is chemically bonded, to obtain an interface named slg-monomer and slg-dimer. Full

geometry optimization of the structures have been followed by a single point at the TD-DFT method, to obtain the absorption spectra.

**Slg-mono**	**Wavelength (o.s.)**	**transition**	**FMO**	
5	601 (0.10)	S → L	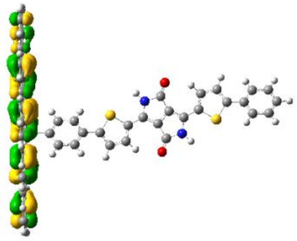	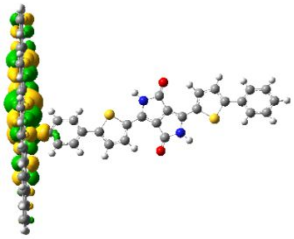
10	495 (0.34)	S → L+2	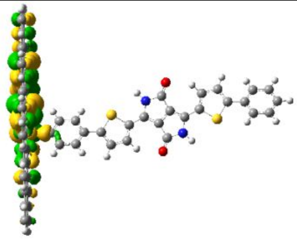	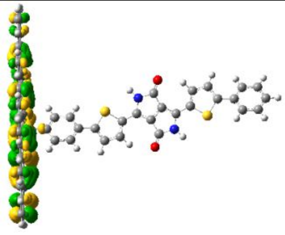
12	472 (1.26)	H-2 → L+1	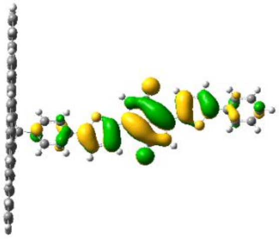	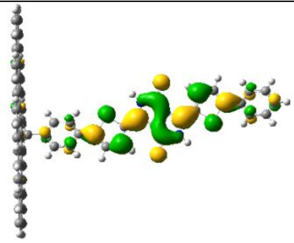
16	447 (0.22)	H-1 → L+1	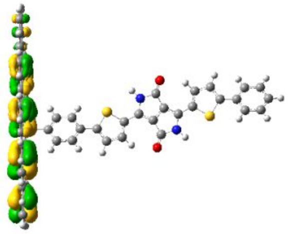	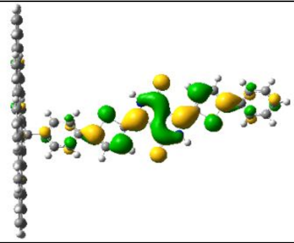
17	440 (0.20)	S → L+3	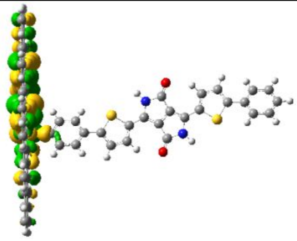	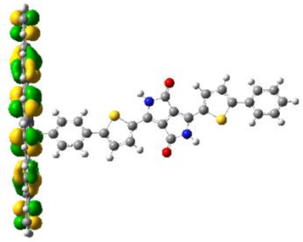
**Slg-dimer**	**Wavelength (o.s.)**	**transition**	**FMO**	

6	600 (0.10)	S → L	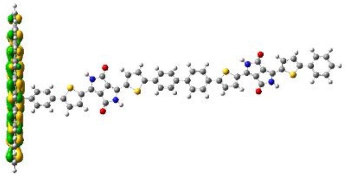	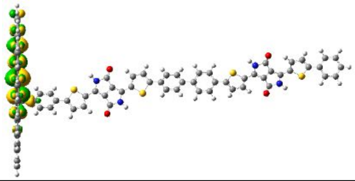
12	496 (0.57)	S → L+3	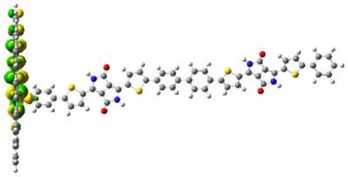	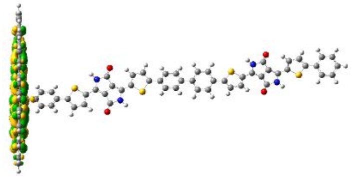
13	483 (2.74)	H-2 → L+2	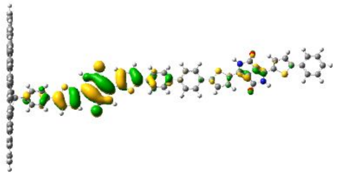	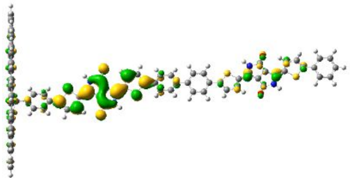

Table S.3: Long wavelength transitions in the SLG-monomer and SLG-dimer systems.

SLG-oligomer-SLG results

**Slg-mono-slg**	**Wavelength (o.s.)**	**transition**	**FMO**	
2	1056 (0.04)	H-2 → L	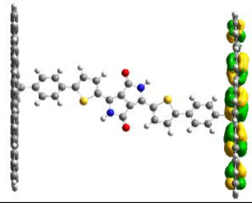	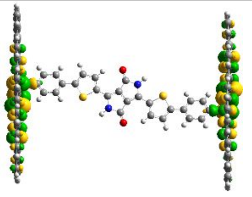
4	886 (0.08)	H → L+1	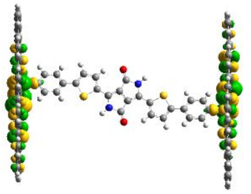	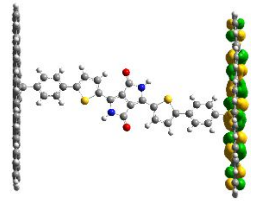
9	680 (0.14)	H → L+3	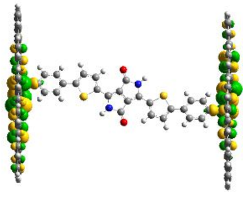	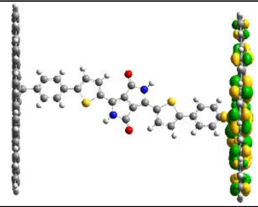
10	659 (0.14)	H → L+6	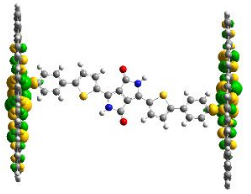	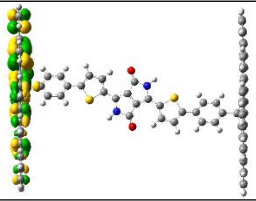

11	652 (0.14)	H-6 → L	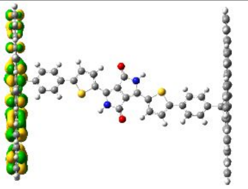	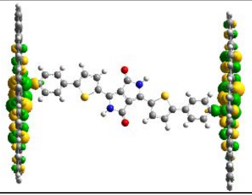
12	641 (0.12)	H-7 →L	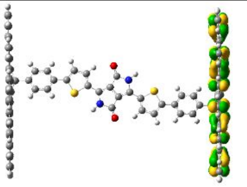	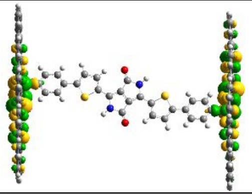
14	595 (0.22)	H →L+5	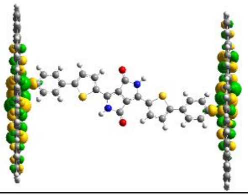	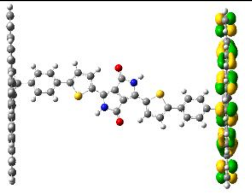
15	585 (0.19)	H →L+7	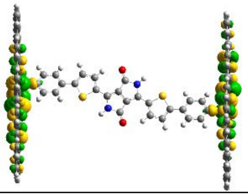	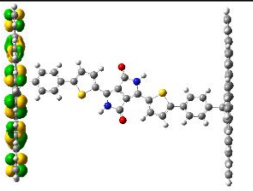
			H-3	L+4
			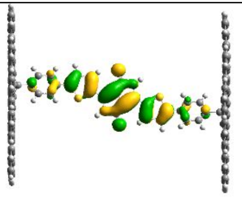	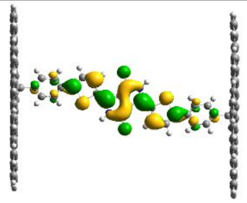
**Slg-dimer-slg**	**Wavelength (o.s.)**	**transition**	**FMO**	
2	995 (0.03)	H-1 → L	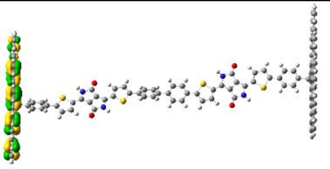	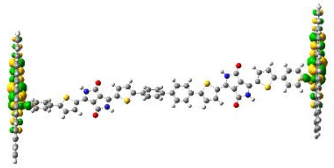
3	981 (0.03)	H-2 → L	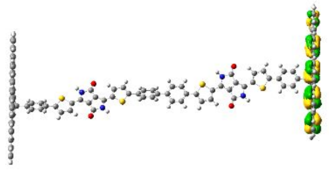	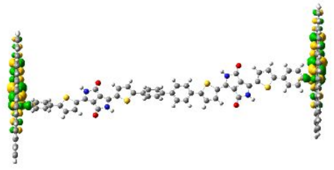
4	816 (0.08)	H → L+1	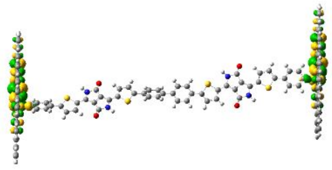	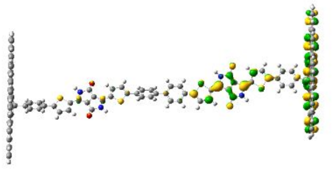

5	793 (0.08)	H → L+3	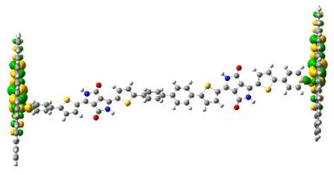	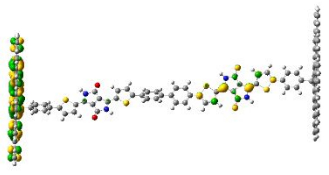
8	667 (0.15)	H → L+5	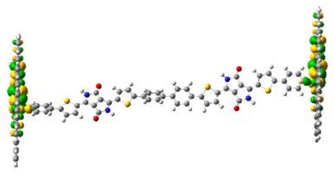	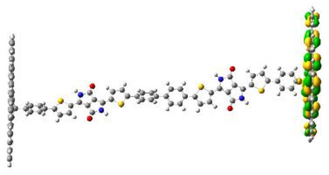
9	658 (0.15)	H-7 → L	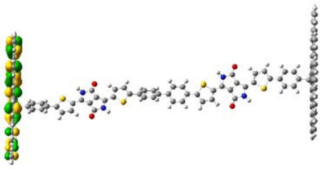	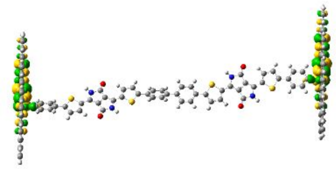
10	655 (0.15)	H → L+6	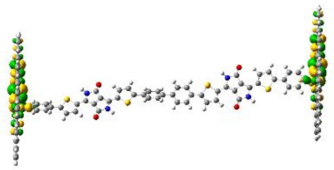	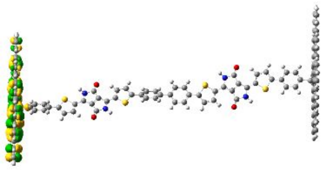
11	648 (0.15)	H-8 → L	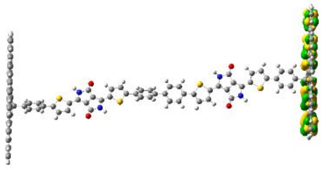	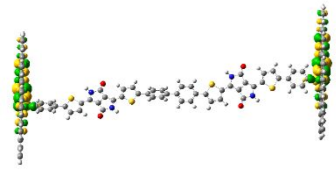
14	592 (0.19)	H → L+7	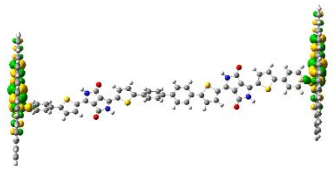	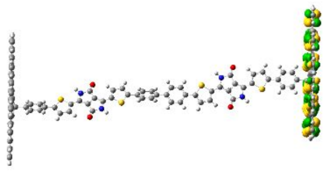

Table S.4: Long wavelength transitions in the SLG-monomer-SLG and SLG-dimer-SLG system.

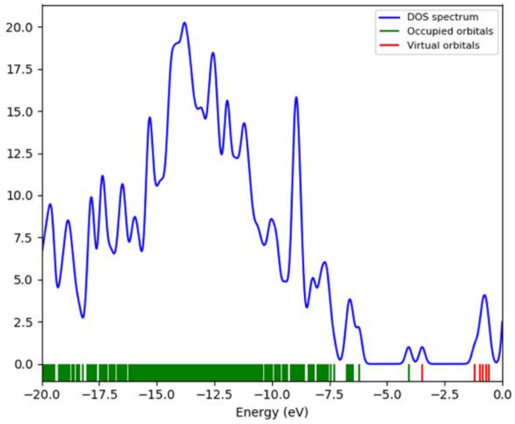

Figure S.20: Density of states (DOS) for SLG-monomer-SLG.

HOMO	SOMO	LUMO

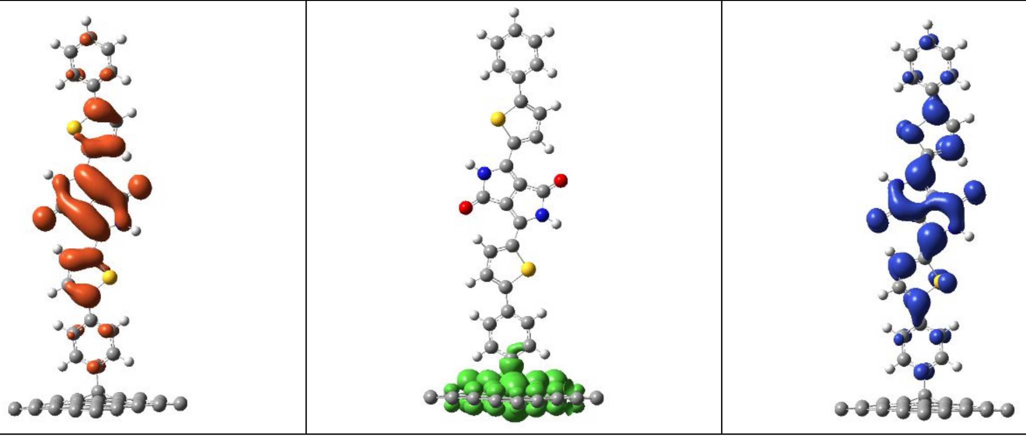

Figure S.21: Frontier molecular orbitals of the nano-SLG-monomer interface from the WF analysis.

Bibliography:

1. Réhault, J.; Maiuri, M.; Oriana, A.; Cerullo, G. Two-dimensional electronic spectroscopy with birefringent wedges. *Rev. Sci. Instrum*. **2014**, 85, 123107.

2. Rätsep, M.; Linnanto, J.; Freiberg, A. Mirror Symmetry and Vibrational Structure in Optical Spectra of Chlorophyll A. *J. Chem. Phys.* **2009**, 130, 194501.

3. Galli, C.; Wynne, K.; LeCours, S. M.; Therien, M. J.; Hochstrasser, R. M. Direct Measurement of Electronic Dephasing Using Anisotropy. *Chem. Phys. Lett.* **1993**, 206, 493–499.

4. Farrow, D. A., Smith, E. R., Qian, W., Jonas, D. M. The polarization anisotropy of vibrational quantum beats in resonant pump-probe experiments: Diagrammatic calculations for square symmetric molecules. *Journal of Chemical Physics* **2008**, 129, 174509.

5. Jonas, D. M., Lang, M. J., Nagasawa, Y., Joo, T., Fleming, G. R. Pump-probe polarization anisotropy study of femtosecond energy transfer within the photosynthetic reaction center of Rhodobacter sphaeroides R26. *Journal of Physical Chemistry* **1996**, 100, 12660–12673.

6. Zheng, M.; Lamberti, F.; Franco, L.; Collini, E.; Fortunati, I.; Bottaro, G.; Daniel, G.; Sorrentino, R.; Minotto, A.; Kukovecz, A. et al. A film-forming graphene/diketopyrrolopyrrole covalent hybrid with far-red optical features: Evidence of photo-stability. *Synthetic Metals* **2019,** 258, 116201.

7. Gustafsson, M. "Spectroscopic Studies of Tissue Using Near-Infrared Raman Microscopy", Master's Thesis, *Lund Reports on Atomic Physics,* LRAP-207, 1997

8. Policht, V. R., Niedringhaus, A., Ogilvie, J. P. Characterization of Vibrational Coherence in Monomeric Bacteriochlorophyll a by Two-Dimensional Electronic Spectroscopy. *The Journal of Physical Chemistry Letters* **2018**, 9, 6631–6637.

9. Rafiq, S., Fu, B., Kudisch, B., Scholes, G. D. Interplay of vibrational wavepackets during an ultrafast electron transfer reaction. *Nature Chemistry* **2021**, 13, 70–76.

10. Senlik, S. S., Policht, V. R., Ogilvie, J. P. Two-Color Nonlinear Spectroscopy for the Rapid Acquisition of Coherent Dynamics. *The Journal of Physical Chemistry Letters* **2015**, 6, 2413–2420.

11. van Stokkum, I. H. M., Larsen, D. S., van Grondelle, R. Global and target analysis of time-resolved spectra. *Biochimica et Biophysica Acta (BBA) – Bioenergetics* **2004**, 1657, 82–104.

12. Volpato, A., Bolzonello, L., Meneghin, E., Collini, E. (2016). Global analysis of coherence and population dynamics in 2D electronic spectroscopy. *Optics Express* **2016**, *24*, 24773.

13. Cooper, D. R., D'Anjou, B., Ghattamaneni, N., Harack, B., Hilke, M., Horth, A., … Yu, V. Experimental Review of Graphene. *ISRN Condensed Matter Physics*, **2012**, 501686.

14. Yang, J., He, Q., Lin, H., Fan, J., Bai, F. Characteristics of Twisted Intramolecular Charge-Transfer State in a Hyperbranched Conjugated Polymer. *Macromolecular Rapid Communications*, **2001**, *22*, 1152.

15. Rafiq, S., Dean, J. C., Scholes, G. D. Observing Vibrational Wavepackets during an Ultrafast Electron Transfer Reaction. *Journal of Physical Chemistry A* **2015**, 119, 11837–11846.
